# Tumor-repopulating cell-derived microparticles elicit cascade amplification of chemotherapy-induced antitumor immunity to boost anti-PD-1 therapy

**DOI:** 10.1038/s41392-023-01658-3

**Published:** 2023-10-25

**Authors:** Nana Bie, Tuying Yong, Zhaohan Wei, Qingle Liang, Xiaoqiong Zhang, Shiyu Li, Xin Li, Jianye Li, Lu Gan, Xiangliang Yang

**Affiliations:** 1 National Engineering Research Center for Nanomedicine, College of Life Science and Technology, Huazhong University of Science and Technology, 430074 Wuhan, China; 2 Key Laboratory of Molecular Biophysics of the Ministry of Education, College of Life Science and Technology, Huazhong University of Science and Technology, 430074 Wuhan, China; 3 Hubei Key Laboratory of Bioinorganic Chemistry and Materia Medica, Huazhong University of Science and Technology, 430074 Wuhan, China

**Keywords:** Drug development, Drug delivery, Drug development, Immunotherapy

## Abstract

Immune checkpoint blockade (ICB) therapy, particularly antibodies targeting the programmed death receptor 1 (PD-1) and its ligand (PD-L1), has revolutionized cancer treatment. However, its efficacy as a standalone therapy remains limited. Although ICB therapy in combination with chemotherapy shows promising therapeutic responses, the challenge lies in amplifying chemotherapy-induced antitumor immunity effectively. This relies on efficient drug delivery to tumor cells and robust antigen presentation by dendritic cells (DCs). Here, we developed tumor-repopulating cell (TRC)-derived microparticles with exceptional tumor targeting to deliver doxorubicin (DOX@3D-MPs) for improve anti-PD-1 therapy. DOX@3D-MPs effectively elicit immunogenic tumor cell death to release sufficient tumor antigens. Heat shock protein 70 (HSP70) overexpressed in DOX@3D-MPs contributes to capturing tumor antigens, promoting their phagocytosis by DCs, and facilitating DCs maturation, leading to the activation of CD8^+^ T cells. DOX@3D-MPs significantly enhance the curative response of anti-PD-1 treatment in large subcutaneous H22 hepatoma, orthotopic 4T1 breast tumor and Panc02 pancreatic tumor models. These results demonstrate that DOX@3D-MPs hold promise as agents to improve the response rate to ICB therapy and generate long-lasting immune memory to prevent tumor relapse.

## Introduction

Immune checkpoint blockade (ICB) therapy, exemplified by anti-programmed death receptor1/programmed death receptor ligand (PD-1/PD-L1) antibodies aiming at reactivating T cell-mediated antitumor immunity to combat tumor cells, represents a potent therapeutic approach.^1,2^ While anti-PD-1/anti-PD-L1 therapy offers the potential for clinical effects akin to a cure,^3^ only fractional patients (20–30%) are expected to profit by anti-PD-1/anti-PD-L1 therapy.^4,5^ Additionally, the development of innate or acquired resistance can ultimately result in cancer progression in patients who initially respond favorably.^6^ Addressing the limitation of therapy resistance represents a substantial challenge for advancing the clinical application of ICB therapy.

The low response rate of ICB therapy is mostly ascribed to the encounter with “immune-cold” tumors distinguished as limited immunogenicity, inadequate or terminal-exhausted tumor-infiltrating lymphocytes and suppressive tumor immune microenvironment (TIM).^7–9^ Numerous approaches striving to convert the “immune-cold” into “immune-hot” tumors have been suggested to augment the effectiveness of ICB therapy, including combination therapies with established treatments like chemotherapy,^10^ radiotherapy,^11^ antiangiogenic therapy,^12^ and targeted therapy.^13^ Chemotherapy, one of the classic strategies for cancer treatment, can directly kill tumor cells while positively regulate antitumor immunity by inducing tumor cells to suffer immunogenic cell death (ICD),^14^ promoting the release of immunomodulatory molecules and tumor antigens and activating dendritic cells (DCs) to increase antigen cross-presentation.^15,16^ Meanwhile, chemotherapy can also trigger the secretion of chemokines like CXCL10, recruiting T cells and memory T cells to TIM.^9,17^ In addition, chemotherapy can improve tumor immunosuppressive microenvironment by reducing the presence of immunosuppressive cells, such as regulatory T cells (Tregs)^18^ and myeloid-derived suppressor cells (MDSCs).^19^ However, conventional chemotherapeutic agents may not achieve the desired ICD effects due to the poor drug delivery to tumor cells.^20^ Besides, the insufficient antigen presentation by DCs results in the inadequate T cell activation.^21^ Therefore, developing efficient chemotherapeutic delivery systems that enhances both tumor cell-targeting and antigen-presentation by DCs holds great promise for advancing the combined therapeutic benefits of ICB therapy and chemotherapy.

Cell-derived extracellular vesicles (EVs), such as microparticles (MPs) and exosomes, exhibit potential advantages as drug transporting systems owing to innate cell or tissue-specific homing capacity, good biosafety and negligible immunogenicity.^22,23^ Meanwhile, cell-derived EVs inherit the signaling molecules of donor cells, exhibiting unique biological features.^24,25^ For example, exosomes secreted by natural killer (NK) cells carry functional NK proteins such as perforin and Fas ligand (FasL), along with cytokines like tumor necrosis factor (TNF)-α to exert cytotoxic effects on tumor cells.^26^ Our previous work has shown that tumor cell-derived exosomes efficiently target tumors by overexpressing intercellular adhesion molecule-1 (ICAM-1, CD54).^27^ Recently, we have developed a drug delivery system based on tumor-repopulating cell (TRC)-derived MPs (3D-MPs) to deliver anticancer drugs for enhanced cancer treatment.^28^ 3D-MPs exhibit unique softness and deformability to achieve increased drug accumulation in tumors, successful diffusion from tumor vessels, effective penetration into deep tumor tissues, and efficient internalization of anticancer drugs into both tumor cells and TRCs owing to the low expression of cytospin-A, a cytoskeleton-related protein. Beyond their improved tumor-targeted drug delivery capabilities, the extent to which 3D-MPs-based drug delivery systems contribute to antitumor immunity necessitates additional investigation.

**Fig. 1:**
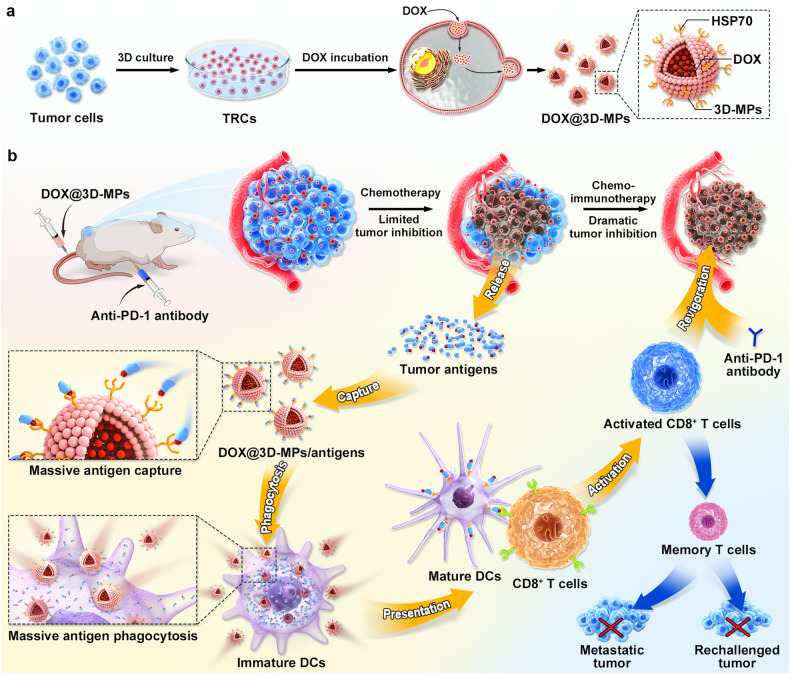
Schematic of DOX@3D-MPs as an efficient enhancer for anti-PD-1 antibody therapy. **a** Schematic illustration of the preparation of DOX@3D-MPs. **b** Schematic illustration of DOX@3D-MPs to boost anti-PD-1 antibody therapy. DOX@3D-MPs efficiently targeted tumor cells and induced ICD of tumor cells to release enough tumor antigens. 3D-MPs assisted capturing tumor antigens to promote their phagocytosis by DCs and the subsequent DC maturation and CD8^+^ T cell activation in a HSP70-dependent manner. Combination of DOX@3D-MPs and anti-PD-1 antibody efficiently hindered tumor growth, generating strong antitumor immune memory to restrain tumor relapse and metastasis

Here, we reveal that 3D-MPs delivering anticancer drug doxorubicin (DOX@3D-MPs) efficiently evoke ICD effect of tumor cells to release massive immunomodulatory factors and tumor antigens. More importantly, the upregulated heat shock protein 70 (HSP70) in DOX@3D-MPs plays an essential role in capturing tumor antigens as well as facilitating their phagocytosis by DCs, which in turn enhances the antigen-presenting capability of DCs and activates tumor-specific CD8^+^ T cells. Consequently, DOX@3D-MPs significantly boost the anticancer efficacy of anti-PD1 antibody, inducing enduring immune memory that effectively curtails tumor recurrence (Fig. 1a, b).

## Results

### DOX@3D-MPs exhibit potent anticancer activity by regulating antitumor immunity

**Fig. 2:**
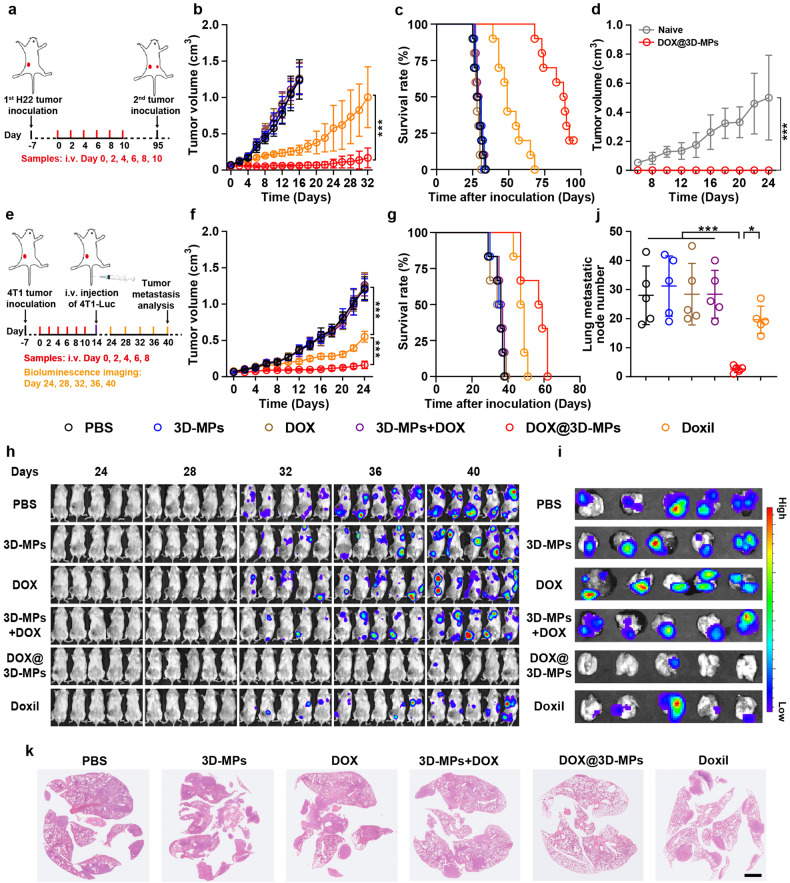
Potent antitumor activity of DOX@3D-MPs. **a**, **e** Schematic timeline for the in vivo pharmacodynamic experiments in subcutaneous H22 (**a**) and orthotopic 4T1 tumor-bearing mice (**e**) after administration of six doses of PBS, 3D-MPs, DOX, 3D-MPs+DOX, DOX@3D-MPs derived from H22 TRCs and 4T1 TRCs at DOX dosage of 0.75 mg kg^−1^ once every other day, respectively, or three doses of high dosage of Doxil at 4 mg kg^−1^ once every 3 days via tail vein. **b**, **f** Tumor volume curves of H22 (**b**) and 4T1 tumor-bearing mice (**f**) after treatments indicated in (**a**, **e**), respectively. (*n* = 10 mice in each group). **c**, **g** Kaplan-Meier survival curves of H22 (**c**) and 4T1 tumor-bearing mice (**g**) after treatments indicated in **a** and **e**, respectively (*n* = 10 mice in each group). **d** Tumor growth curves of naive mice or DOX@3D-MPs-cured mice indicated in (**a**) after the second inoculation with H22 cells (2 × 10^6^ cells). (*n* = 10 for naive mice, *n* = 2 for DOX@3D-MPs-cured mice). **h** In vivo bioluminescence images monitoring the growth and spreading of intravenously inoculated 4T1-Luc cells in orthotopic 4T1 tumor-bearing mice after treatments indicated in (**e**). **i** Ex vivo bioluminescence images of lung metastatic nodes at 40 days after treatments indicated in (**e**). **j** Numbers of lung metastatic nodes of 4T1 tumor-bearing mice at 40 days after treatments indicated in (**e**). (*n* = 5 mice in each group). **k** Representative H&E staining images of the metastatic lungs at 40 days after treatments indicated in (**e**). Scale bar: 2 mm. Data are shown as means ± s.d. *P < 0.05, ***P < 0.001

Our previous work has shown that DOX@3D-MPs exhibited strong anticancer activity.^28^ To further confirm the anticancer effects of DOX@3D-MPs, mice bearing subcutaneous hepatoma H22 tumors and orthotopic pancreatic Panc02 tumors were subjected to intravenous administration of PBS, 3D-MPs, DOX, combination of 3D-MPs and DOX (denoted as 3D-MPs+DOX), DOX@3D-MPs or higher-dosed Doxil (Fig. 2a and Supplementary Fig. 1a). Coherently, DOX@3D-MPs showed the best anticancer capacity, which was noticeably evidenced by the lowest tumor size (Fig. 2b and Supplementary Fig. 2a-f) or weight (Supplementary Fig. 1b, c and Supplementary Fig. 3a, b) compared with other groups in both H22 and Panc02 tumor-bearing mice. In addition, DOX@3D-MPs obviously reduced the tumor metastatic node numbers in the intestines of Panc02 tumor-bearing mice (Supplementary Fig. 1d, e). Meanwhile, DOX@3D-MPs-treated mice have the longest survival time when compared to other groups (Fig. 2c and Supplementary Fig. 1f). Especially, two of ten H22 tumor-bearing mice had complete tumor ablation after treatment with DOX@3D-MPs (Supplementary Fig. 2e). When these two mice were subjected to a second inoculation with H22 cells, total tumor rejection was observed although all the naïve mice in control group that had been injected with the identical amounts of H22 cells had tumor formation and continued tumor growth (Fig. 2d). Moreover, complete 4T1 tumor formation was detected in these two mice after inoculation of 4T1 cells (Supplementary Fig. 4), suggesting that DOX@3D-MPs might efficiently inhibit tumor recurrence through regulating the tumor-specific immune response. Post-treatment of DOX@3D-MPs did not cause detectable toxicity from the body weight variation tendency and serological analysis (Supplementary Figs. 5 and 6a–d) although high dosage of Doxil induced toxicity, revealing the good biosafety of DOX@3D-MPs.

To further confirm that DOX@3D-MPs possessed good anticancer activity, the mice inoculated with orthotopic breast 4T1 tumors were administrated with PBS, 3D-MPs, DOX, 3D-MPs+DOX, DOX@3D-MPs or high dose of Doxil via tail vein. To establish tumor model to simulate tumor metastasis and recurrence, the 4T1 tumor-bearing mice were inoculated with luciferase-expressing 4T1 (4T1-Luc) cells via tail vein after treatment (Fig. 2e). Coherently, DOX@3D-MPs markedly hampered the growth of primary orthotopic 4T1 tumors (Fig. 2f and Supplementary Fig. 7) and elongated the survival time of 4T1 tumor-bearing mice (Fig. 2g). In vivo bioluminescence observation revealed that the bioluminescence signal designating tumor recurrence and metastasis grew stronger over time in these mice except those received DOX@3D-MPs treatment (Fig. 2h). Simultaneously, ex vivo bioluminescence imaging of metastatic lung tissues exhibited the weakest bioluminescence signal in lungs from mice received DOX@3D-MPs treatment (Fig. 2i), revealing efficient inhibition of the formation of 4T1-Luc tumors in lungs to suppress tumor recurrence and metastasis. In addition, remarkably lower numbers of metastatic nodes in lungs were observed in the mice administrated by DOX@3D-MPs, as evidenced by the quantification of node numbers (Fig. 2j) and histopathological analysis of lung tissue through H&E staining (Fig. 2k). Considering that the inhibition of 4T1-Luc tumor formation occurs after DOX@3D-MPs administration, the DOX@3D-MPs-induced inhibition in tumor recurrence and metastasis might be modulated by antitumor immunity. To further clarify that DOX@3D-MPs-induced antitumor effects was regulated by antitumor immunity, the antitumor activity of DOX@3D-MPs was evaluated in H22 tumor model established by nude mice (Supplementary Fig. 8a). No significant tumor inhibition and elongated survival time were detected in tumor-inoculated nude mice received DOX@3D-MPs administration (Supplementary Fig. 8b, c). Meanwhile, blockade of whole CD8^+^ T cells with anti-CD8 antibody, but not NK cells noticeably hampered the antitumor activity of DOX@3D-MPs (Supplementary Fig. 8d–f), confirming that DOX@3D-MPs-induced antitumor activity was mediated by CD8^+^ T cell-involved antitumor immunity.

### DOX@3D-MPs efficiently evoke antitumor immunity in vitro

**Fig. 3:**
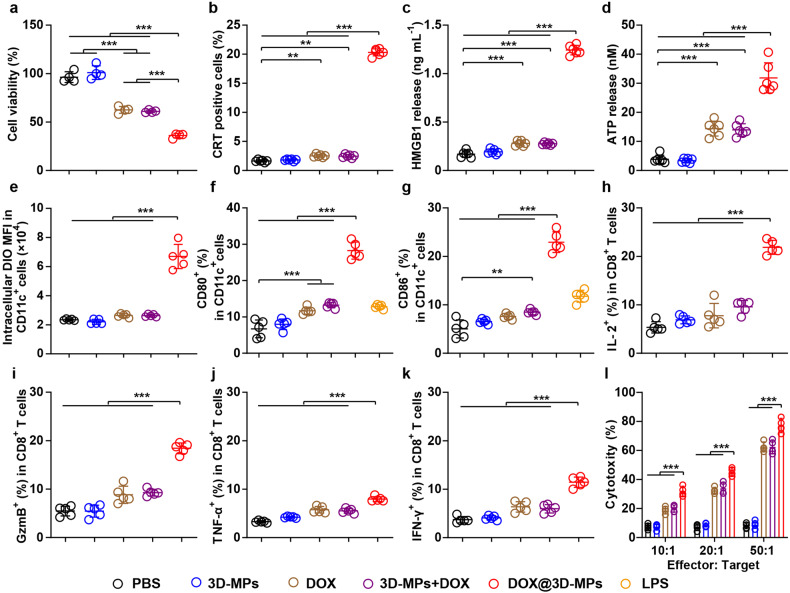
In vitro antitumor immunity induced by DOX@3D-MPs. **a** Cell viability of H22 cells at 24 h after treatment with PBS, 3D-MPs, DOX, 3D-MPs+DOX or DOX@3D-MPs derived from H22 TRCs at DOX concentration of 1 μg mL^−1^. (*n* = 4). **b** CRT^+^ cell ratios of H22 cells at 12 h after treatments indicated in **a** by flow cytometry. (*n* = 6). **c**, **d** HMGB1 (**c**) and ATP release (**d**) from H22 cells at 12 h after treatments indicated in (**a**). (*n* = 6). **e** Intracellular DiO mean fluorescence intensity (MFI) in CD11c^+^ BMDCs at 12 h after immature BMDCs were co-cultured with the DiO-labeled H22 cells treated as indicated in **a** by flow cytometry. (*n* = 5). **f**, **g** Percentages of CD80^+^ (**f**) and CD86^+^ (**g**) cells in CD11c^+^ BMDCs at 12 h after immature BMDCs were co-cultured with H22 cells treated as indicated in **a** by flow cytometry. LPS (100 ng mL^−1^) was used as a positive control. (*n* = 5). **h**–**k** Percentages of IL-2^+^ (**h**), GzmB^+^ (**i**), TNF-α^+^ (**j**) and IFN-γ^+^ (**k**) cells in CD8^+^ T cells after CD3^+^ T cells were incubated with the matured BMDCs treated as indicated in **f** for 3 days by flow cytometry. (*n* = 5). **l** Cytotoxicity of activated T cells (effector cells) against H22 cells (target cells) at 24 h after the activated T cells treated as indicated in **h** were incubated with H22 cells at different effector/target ratios by LDH assay. (*n* = 3). Data are shown as means ± s.d. **P < 0.01, ***P < 0.001

Certain chemotherapeutic drugs like DOX can elicit ICD in cancer cells to trigger DC maturation and activate antitumor immunity largely mediated by tumor-specific T cells.^29^ As expected, DOX@3D-MPs exhibited stronger cytotoxicity against H22 (Fig. 3a), 4T1 (Supplementary Fig. 9a) and ovalbumin (OVA)-expressing B16 (B16-OVA) cells (Supplementary Fig. 10a) compared with free DOX and 3D-MPs+DOX, which might be owing to the higher cellular uptake (Supplementary Fig. 11a–c). Consistently, the stronger exposure of calreticulin (CRT) (Fig. 3b and Supplementary Figs. 9b and 10b), and more release of high mobility group box 1 (HMGB1) (Fig. 3c and Supplementary Fig. 9c) and secretion of adenosine triphosphate (ATP) (Fig. 3d and Supplementary Fig. 9d), the typical signaling molecules involved in ICD,^30^ were observed in H22, 4T1 and B16-OVA cells treated by DOX@3D-MPs as compared to those treated by free DOX and 3D-MPs+DOX, suggesting that DOX@3D-MPs evoked stronger ICD effects in tumor cells. When the above treated H22, 4T1 or B16-OVA cells labeled with DiO were co-cultured with bone marrow-derived dendritic cells (BMDCs), more DOX@3D-MPs-treated H22, 4T1 or B16-OVA cells were phagocytosed by BMDCs compared with other treatments (Fig. 3e and Supplementary Figs. 9e and 10c). Meanwhile, the stronger expressions of costimulatory molecules CD80 (Fig. 3f and Supplementary Figs. 9f and 10d) and CD86 (Fig. 3g and Supplementary Figs. 9g and 10e), the indicators for the maturation of DCs, were observed in BMDCs co-incubated with DOX@3D-MPs-treated tumor cells, demonstrating that DOX@3D-MPs-triggered ICD efficiently evoked DC maturation, even better than a ligand of toll-like receptor 4 lipopolysaccharide (LPS), a positive control to induce DC maturation by binding with toll-like receptors (TLRs) on DCs.^31,32^ In addition, the highest major histocompatibility complex (MHC)-I presentation of OVA (H-2Kb OVA^+^) was detected in BMDCs co-incubated with DOX@3D-MPs-treated B16-OVA cells (Supplementary Fig. 10f), suggesting the efficient presentation of antigens from DOX@3D-MPs-treated tumor cells by BMDCs. Furthermore, flow cytometric analysis showed that when naïve T cells derived from spleens were co-incubated with the above treated BMDCs, the highest percentages of effector molecules interleukin (IL)-2- (Fig. 3h and Supplementary Fig. 9h), granzyme B (GzmB)- (Fig. 3i and Supplementary Fig. 9i), TNF-α- (Fig. 3j and Supplementary Fig. 9j) and interferon (IFN)-γ-generating CD8^+^ T cells (Fig. 3k and Supplementary Fig. 9k) were observed in T cells from DOX@3D-MPs-treated group, indicating that BMDCs matured by DOX@3D-MPs-treated tumor cells displayed the most robust capability for activating CD8^+^ T cells. Correspondingly, the above CD8^+^ T cells that were activated by the BMDCs from DOX@3D-MPs-treated group evoked the strongest cytotoxicity against the respective H22, 4T1 or B16-OVA cells (Fig. 3l and Supplementary Figs. 9l and 10g), confirming that DOX@3D-MPs-triggered ICD efficiently activate tumor-specific CD8^+^ T cells to elicit antitumor immune response.

### DOX@3D-MPs efficiently elicit antitumor immunity in vivo

**Fig. 4:**
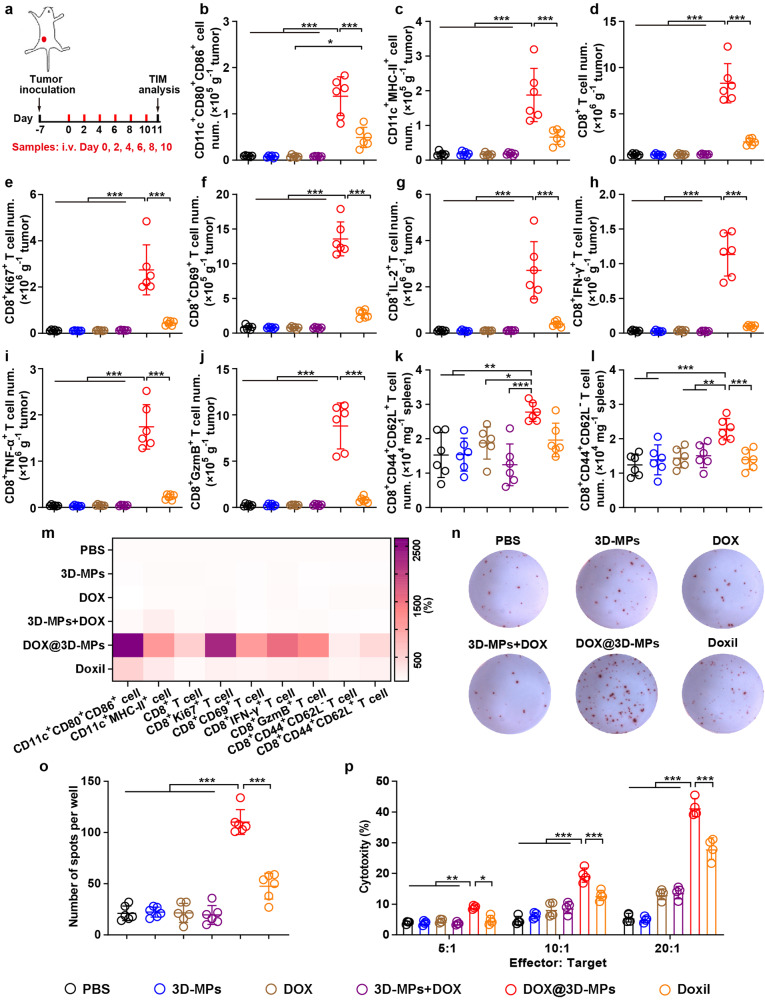
DOX@3D-MPs-induced enhanced antitumor immunity in H22 and 4T1 tumor-bearing mice. **a** Schematic timeline for tumor immune microenvironment analysis in H22 and 4T1 tumor-bearing mice after administration of six doses of PBS, 3D-MPs, DOX, 3D-MPs+DOX, DOX@3D-MPs derived from H22 TRCs and 4T1 TRCs at DOX dosage of 0.75 mg kg^−1^ once every other day, respectively, or three doses of high dosage of Doxil at 4 mg kg^−1^ once every 3 days via tail vein. **b**–**j** Numbers of CD11c^+^CD80^+^CD86^+^ (**b**), CD11c^+^MHC-II^+^ (**c**), CD8^+^ T (**d**), CD8^+^Ki67^+^ T (**e**), CD8^+^CD69^+^ T (**f**), CD8^+^IL-2^+^ T (**g**), CD8^+^IFN-γ^+^ T (**h**), CD8^+^TNF-α^+^ T (**i**), and CD8^+^GzmB^+^ T (**j**) cells in tumor tissues of H22 tumor-bearing mice at 11 days after treatments indicated in **a**. (n = 6 mice in each group). **k**, **l** Numbers of CD8^+^CD44^+^CD62L^+^ T (**k**) and CD8^+^CD44^+^CD62L^-^ T cells (**l**) in the spleens of H22 tumor-bearing mice at 11 days after treatments indicated in (**a**). (*n* = 6 mice in each group). **m** Relative numbers of CD11c^+^CD80^+^CD86^+^, CD11c^+^MHC-II^+^, CD8^+^ T, CD8^+^Ki67^+^ T, CD8^+^CD69^+^ T, CD8^+^IFN-γ^+^ T and CD8^+^GzmB^+^ T cells in tumor tissues, and CD8^+^CD44^+^CD62L^+^ T and CD8^+^CD44^+^CD62L^-^ T cells in the spleens of orthotopic 4T1 tumor-bearing mice at 11 days after treatments indicated in (**a**). (*n* = 6 mice in each group). **n**, **o** Representative images (**n**) and numbers (**o**) of IFN-γ immune spots from 4T1 cell debris-restimulated splenocytes isolated from orthotopic 4T1 tumor-bearing mice after treatments indicated in (**a**) by ELISpot assay. (*n* = 6 mice in each group). **p** Cytotoxicity of T cells (effector cells) against 4T1 cells (target cells) at 24 h after T cells isolated from 4T1 cell debris-restimulated splenocytes of orthotopic 4T1 tumor-bearing mice treated as indicated in **a** were incubated with 4T1 cells at different effector/target ratios by LDH assay. (*n* = 3). Data are shown as means ± s.d. *P < 0.05, **P < 0.01, ***P < 0.001

To further investigate DOX@3D-MPs-induced antitumor immunity, the TIM was first evaluated in subcutaneous H22 tumor models after administrated with six doses of PBS, 3D-MPs, DOX, 3D-MPs+DOX, DOX@3D-MPs or higher-dosed Doxil intravenously (Fig. 4a). As expected, DOX@3D-MPs treatment remarkably increased the amounts of CD11c^+^CD80^+^CD86^+^ (Fig. 4b and Supplementary Fig. 12) and CD11c^+^MHC-II^+^ cells (Fig. 4c) in tumor tissues, revealing that DOX@3D-MPs efficiently promoted intratumor DC maturation. Furthermore, the amounts of CD8^+^ T (Fig. 4d and Supplementary Fig. 12), but not CD4^+^ T cells (Supplementary Figs. 12 and 13a) were significantly upregulated in DOX@3D-MPs-treated group. Meanwhile, the amounts of proliferated CD8^+^Ki67^+^ T (Fig. 4e), and functional CD8^+^CD69^+^ T (Fig. 4f), CD8^+^IL-2^+^ T (Fig. 4g), CD8^+^IFN-γ^+^ T (Fig. 4h), CD8^+^TNF-α^+^ T (Fig. 4i) and CD8^+^GzmB^+^ T cells (Fig. 4j) were the highest in tumor tissues of mice received DOX@3D-MPs treatment, indicating that DOX@3D-MPs exhibited the strong ability to activate antitumor immunity. In addition, treatment with DOX@3D-MPs markedly reduced the amounts of immunosuppressive cells like MDSCs (Supplementary Fig. 13b) and Tregs (Supplementary Figs. 12 and 13c), suggesting the reshaped TIM by DOX@3D-MPs. Similar phenomenon was observed in relevant immune cells derived from tumor draining lymph nodes (TDLNs) (Supplementary Fig. 14a–l) and spleens (Supplementary Fig. 15a–l) of mice received DOX@3D-MPs administration. Importantly, DOX@3D-MPs treatment significantly enhanced the amounts of central memory T (Tcm) cells (CD8^+^CD44^+^CD62L^+^ T cells) and effector memory T (Tem) cells (CD8^+^CD44^+^CD62L^-^ T cells) in the spleens (Fig. 4k, l) and TDLNs (Supplementary Fig. 16a, b) of H22 tumor-inoculated mice compared with mice administrated with other treatments, demonstrating that DOX@3D-MPs might effectively induce antitumor immune memory. The DOX@3D-MPs-induced enhanced antitumor immunity and improved TIM were additionally verified in orthotopic 4T1 tumor model (Fig. 4m and Supplementary Figs. 17a–i and 18a–j). Moreover, when T cells isolated from the spleens of the above treated 4T1 tumor-inoculated mice were subjected to restimulation of 4T1 cell lysates, ELISpot assay showed markedly higher frequency of IFN-γ-secreting cells among T cells of mice received DOX@3D-MPs treatment than other treatments (Fig. 4n, o). In addition, T cells derived from the splenocytes of mice received DOX@3D-MPs treatment exerted stronger cytotoxicity against 4T1 cells after re-stimulation of 4T1 cell lysates (Fig. 4p), further confirming that DOX@3D-MPs efficiently triggered tumor-specific antitumor immune memory.

### DOX@3D-MPs induce antitumor immune response in a HSP70-dependent manner

**Fig. 5:**
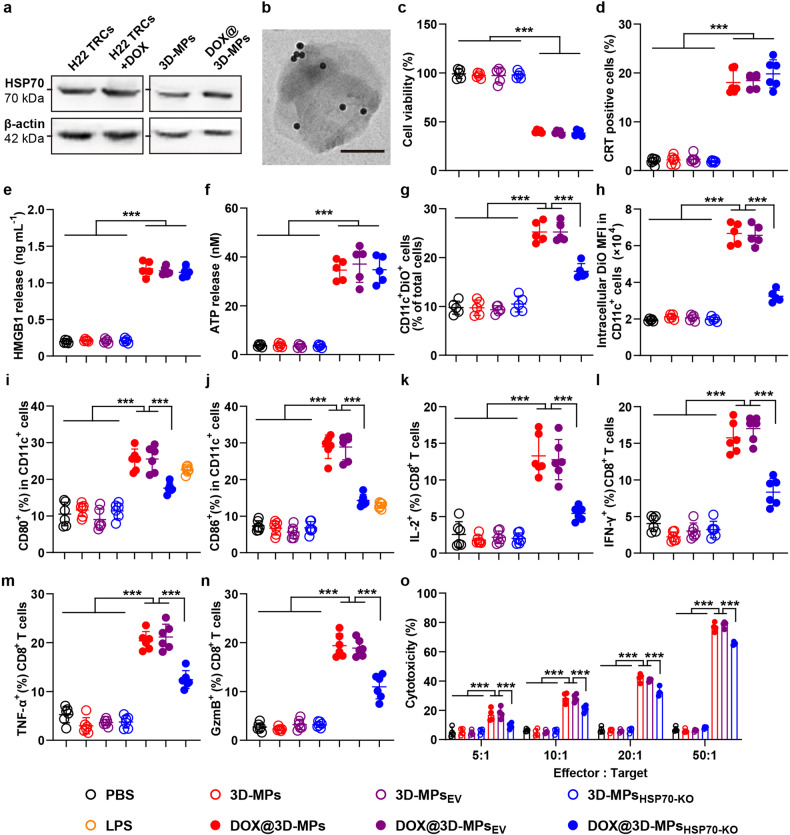
DOX@3D-MPs-induced in vitro antitumor immunity in a HSP70-dependent manner. **a** HSP70 expression in H22 TRCs, H22 TRCs treated with 200 μg mL^−1^ DOX for 6 h, 3D-MPs and DOX@3D-MPs derived from H22 TRCs by western blot analysis. **b** Representative immune TEM images of HSP70 in DOX@3D-MPs. Scale bar: 200 nm. **c** Cell viability of H22 cells at 24 h after treatment with PBS, 3D-MPs, 3D-MPs_EV_, 3D-MPs_HSP70-KO_, DOX@3D-MPs, DOX@3D-MPs_EV_ or DOX@3D-MPs_HSP70-KO_ derived from H22 TRCs at DOX concentration of 1 μg mL^−1^. (*n* = 5). **d** CRT^+^ cell ratios of H22 cells at 12 h after treatments indicated in **c** by flow cytometry. (*n* = 6). **e**, **f** HMGB1 (**e**) and ATP release (**f**) from H22 cells at 12 h after treatments indicated in (**c**). (*n* = 5). **g**, **h** Ratio of DiO^+^ cells (**g**) and intracellular DiO MFI (**h**) in CD11c^+^ BMDCs at 12 h after the immature BMDCs were co-cultured with DiO-labeled H22 cells treated as indicated in **c** by flow cytometry. (*n* = 5). **i**, **j** Percentages of CD80^+^ (**i**) and CD86^+^ (**j**) cells in CD11c^**+**^ BMDCs at 12 h after the immature BMDCs were co-cultured with H22 cells treated as indicated in **c** by flow cytometry. LPS (100 ng mL^−1^) was used as a positive control. (*n* = 5). **k**–**n** Percentages of IL-2^+^ (**k**), IFN-γ^+^ (**l**), TNF-α^+^ (**m**), and GzmB^+^ (**n**) cells in CD8^+^ T cells after CD3^+^ T cells were incubated with the matured BMDCs treated as indicated in **i** for 3 days by flow cytometry. (*n* = 6). **o** Cytotoxicity of activated T cells (effector cells) against H22 cells (target cells) at 24 h after activated T cells treated as indicated in (**k**) were incubated with H22 cells at different effector/target ratios by LDH assay. (*n* = 3). Data are presented as means ± s.d. ***P < 0.001

HSP70, functioning as a molecular chaperone and ICD marker, participates in the antigen capture and presentation by DCs to promote antitumor immunity^33,34^ As expected, DOX treatment significantly enhanced the expression of HSP70 in H22 TRCs by western blot analysis (Fig. 5a). Correspondingly, HSP70 was more upregulated in DOX@3D-MPs compared to that in 3D-MPs (Fig. 5a). HSP70 was mainly distributed in the outer layer of DOX@3D-MPs by immune transmission electron microscope (TEM) analysis (Fig. 5b). To determine whether HSP70 was involved in DOX@3D-MPs-induced enhanced antitumor immunity, H22 TRCs stably knocking out HSP70 (denoted as TRC_HSP70-KO_) were established by using the CRISPR/Cas9 system, which led to the downregulation of HSP70 in 3D-MPs released by these cells (denoted as 3D-MPs_HSP70-KO_) (Supplementary Fig. 19). No significant cytotoxic difference was detected in H22 cells treated by DOX@3D-MPs_HSP70-KO_ compared with that treated by DOX@3D-MPs and DOX@3D-MPs produced by empty vector-stably expressed H22 TRCs (denoted as DOX@3D-MPs_EV_) (Fig. 5c). Consistently, H22 cells subjected to DOX@3D-MPs_HSP70-KO_ suffered similar level of ICD to that evoked by DOX@3D-MPs and DOX@3D-MPs_EV_, as evidenced by similar CRT exposure (Fig. 5d), HMGB1 release (Fig. 5e) and ATP secretion (Fig. 5f) in these treated groups, suggesting that HSP70 did not significantly affect DOX@3D-MPs-induced cytotoxicity and ICD effects in tumor cells. However, lower H22 cells treated with DOX@3D-MPs_HSP70-KO_ were phagocytosed by BMDCs than DOX@3D-MPs-treated group, while there were no significant difference observed between DOX@3D-MPs- and DOX@3D-MPs_EV_-treated groups (Fig. 5g, h). Correspondingly, the expressions of CD80 (Fig. 5i) and CD86 (Fig. 5j) in BMDCs incubated with DOX@3D-MPs_HSP70-KO_-treated H22 cells were significantly lower than those of DOX@3D-MPs- and DOX@3D-MPs_EV_-treated groups, suggesting that knocking out HSP70 in 3D-MPs significantly decreased BMDC maturation triggered by DOX@3D-MPs-induced ICD. Moreover, the percentages of IL-2- (Fig. 5k), IFN-γ- (Fig. 5l), TNF-α- (Fig. 5m) and GzmB-producing CD8^+^ T cells (Fig. 5n) were significantly lower in CD8^+^ T cells co-incubated with BMDCs matured by H22 cells treated by DOX@3D-MPs_HSP70-KO_ than those of DOX@3D-MPs- and DOX@3D-MPs_EV_-treated groups, resulting in lower cytotoxicity mediated by activated CD8^+^ T cells against H22 cells (Fig. 5o). Above results indicated that HSP70 in 3D-MPs might contribute to the phagocytosis of tumor antigen released from DOX@3D-MPs-treated tumor cells by BMDCs to increase BMDC maturation and CD8^+^ T cell activation. To further confirm the HSP70-regulated antigen capture and phagocytosis by BMDCs, 3D-MPs, 3D-MPs_EV_ or 3D-MPs_HSP70-KO_ were incubated with FITC-labeled OVA (a model antigen) or DiO-labeled cell debris, and then the antigen capture by 3D-MPs was determined by flow cytometry. Consistently, FITC (Supplementary Fig. 20a) or DiO fluorescence (Supplementary Fig. 20b) was significantly lower in 3D-MPs_HSP70-KO_ compared with 3D-MPs and 3D-MPs_EV_. Moreover, BMDCs phagocytosed less FITC-labeled OVA or DiO-labeled cell debris pretreated with 3D-MPs_HSP70-KO_ (Supplementary Fig. 20c, d) or HSP70 antibody pretreated-3D-MPs (Supplementary Fig. 21a, b), confirming that HSP70 promoted tumor antigen capture by 3D-MPs and enhanced antigen phagocytosis by BMDCs. Here, we noticed that DOX@3D-MPs did not remarkably affect cell viability of BMDCs at the used DOX concentration (Supplementary Fig. 22), suggesting that the simultaneous phagocytosis of DOX@3D-MPs accompanied by the HSP70-captured tumor antigens did not generate toxicity to BMDCs.

**Fig. 6:**
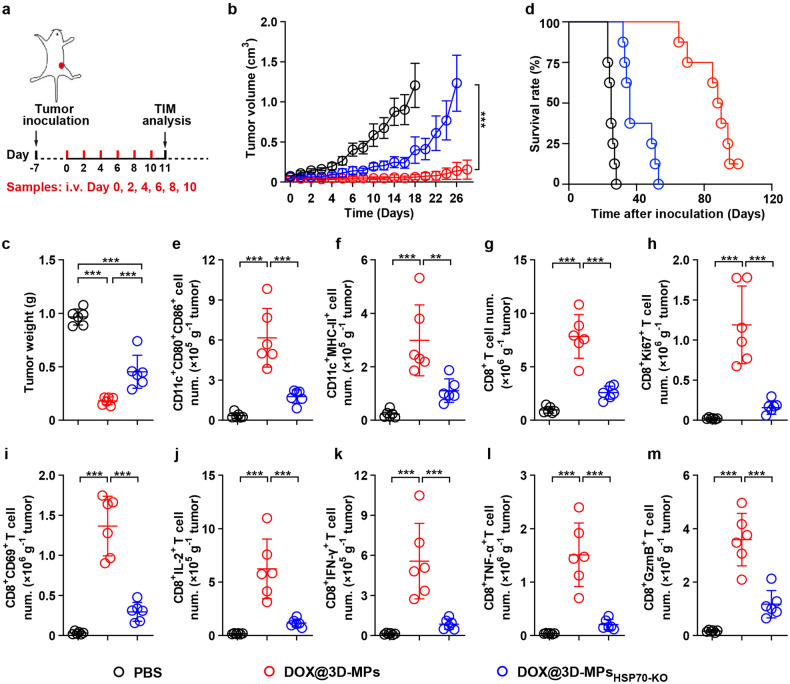
DOX@3D-MPs-induced anticancer activity and enhanced antitumor immunity in a HSP70-dependent manner in vivo. **a** Schematic timeline for anticancer activity and tumor immune microenvironment analysis in H22 tumor-bearing mice after intravenous injection of PBS, DOX@3D-MPs or DOX@3D-MPs_HSP70-KO_ at DOX dosage of 0.75 mg kg^−1^ once every other day for 6 times. **b**, **c** Tumor volume curves (**b**) and tumor weights (**c**) of H22 tumor-bearing mice after treatments indicated in (**a**). (*n* = 8 and 6 mice per group for **b** and **c**, respectively). **d** Kaplan–Meier survival plots of H22 tumor-bearing mice after treatments indicated in (**a**). (*n* = 8 mice in each group). **e**–**m** Numbers of CD11c^+^CD80^+^CD86^+^ (**e**), CD11c^+^MHC-II^+^ (**f**), CD8^+^ T (**g**), CD8^+^Ki67^+^ T (**h**), CD8^+^CD69^+^ T (**i**), CD8^+^IL-2^+^ T (**j**), CD8^+^IFN-γ^+^ T (**k**), CD8^+^TNF-α^+^ T (**l**) and CD8^+^GzmB^+^ T (**m**) cells in tumor tissues of H22 tumor-bearing mice at 11 days after treatments indicated in (**a**). (*n* = 6 mice in each group). Data are shown as means ± s.d. **P < 0.01, ***P < 0.001

To further evaluate the effects of HSP70 on the DOX@3D-MPs-induced antitumor activity and immune response, mice bearing subcutaneous H22 tumors were administrated with PBS, DOX@3D-MPs or DOX@3D-MPs_HSP70-KO_ intravenously (Fig. 6a). As expected, DOX@3D-MPs markedly delayed the growth of H22 tumors, as indicated by the lower tumor volume (Fig. 6b) and weight (Fig. 6c) as well as the longer survival time (Fig. 6d) of tumor-inoculated mice in comparison with mice treated by PBS. Knocking out HSP70 in 3D-MPs significantly hampered the antitumor effects of DOX@3D-MPs (Fig. 6b-d), indicating the vital function of HSP70 in DOX@3D-MPs-triggered anticancer activity. Consistently, knocking out HSP70 in 3D-MPs significantly abrogated the DOX@3D-MPs-evoked increase in the amounts of CD11c^+^CD80^+^CD86^+^ (Fig. 6e), CD11c^+^MHC-II^+^ (Fig. 6f), CD8^+^ T (Fig. 6g), proliferative CD8^+^Ki67^+^ T (Fig. 6h), and activated CD8^+^CD69^+^ T (Fig. 6i), CD8^+^IL-2^+^ T (Fig. 6j), CD8^+^IFN-γ^+^ T (Fig. 6k), CD8^+^TNF-α^+^ T (Fig. 6l) and CD8^+^GzmB^+^ T cells (Fig. 6m) within tumor tissues, suggesting that HSP70 in 3D-MPs played an important role in DOX@3D-MPs-induced DC maturation as well as activation of CD8^+^ T cells within TIM. HSP70 involvement in DOX@3D-MPs-induced antitumor immunity was additionally estimated in TDLNs (Supplementary Fig. 23a–i) and spleens (Supplementary Fig. 24a–i) of H22 tumor-bearing mice. Overall, these data demonstrate that the strong antitumor capacity of DOX@3D-MPs might be attributed to the overexpressed HSP70 in 3D-MPs, resulting in enhanced capture of DOX@3D-MPs-induced tumor antigens and the subsequent increased phagocytosis by DCs, DC maturation as well as CD8^+^ T cell activation for improved antitumor immunity.

### DOX@3D-MPs markedly boost antitumor efficiency of anti-PD-1 antibody

**Fig. 7:**
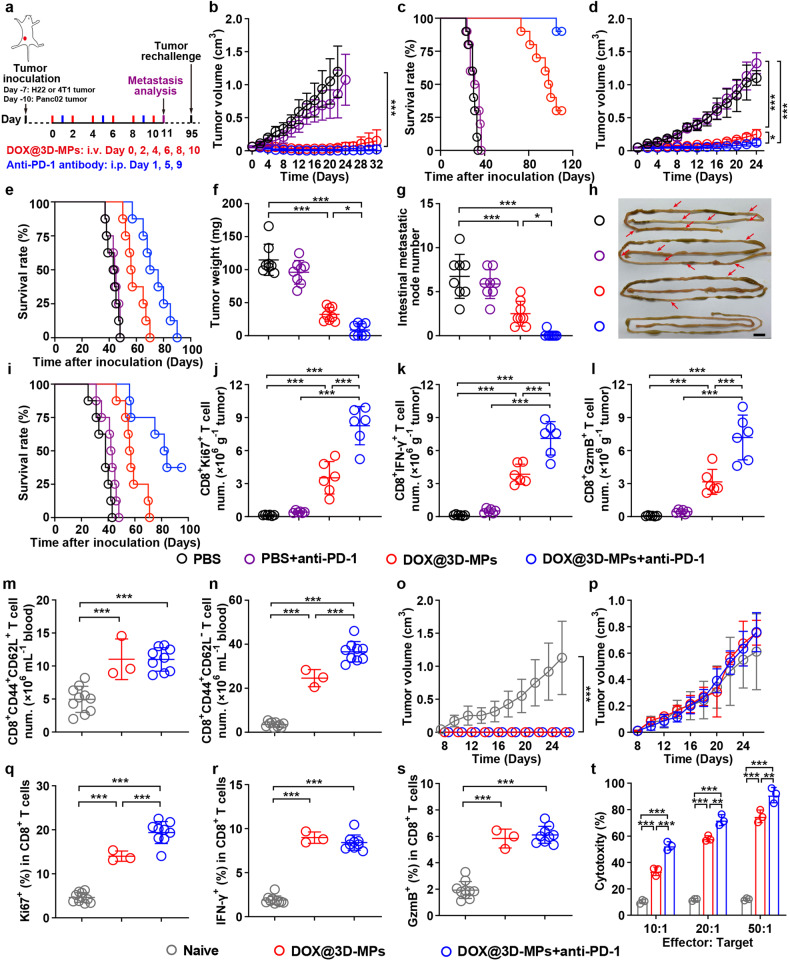
Potent antitumor activity of the combination of DOX@3D-MPs and anti-PD-1 antibody. **a** Schematic timeline for the antitumor experiments in H22, 4T1 and Panc02 tumor-bearing mice after administration of six doses of PBS or DOX@3D-MPs derived from H22 TRCs, 4T1 TRCs and Pan02 TRCs at DOX dosage of 0.75 mg kg^−1^ once every other day with or without of intraperitoneal injection of anti-PD-1 antibody at the dosage of 5 mg kg^−1^ every 4 days for three times, respectively. **b**, **d** Tumor volume curves of H22 (**b**) and 4T1 tumor-bearing mice (**d**) after treatments indicated in **a**. (*n* = 8 mice in each group). **c**, **e**, **i** Kaplan-Meier survival plots of H22 (**c**), 4T1 (**e**) and Panc02 tumor-bearing mice (**i**) after treatments indicated in (**a**) (*n* = 10 mice in each group for (**c**) and *n* = 8 mice in each group for (**e** and **i**)). **f** Tumor weights of Panc02 tumor-bearing mice at 11 days after treatments indicated in (**a**). (*n* = 8 mice in each group). **g**, **h** Numbers (**g**) and representative images (**h**) of intestine metastatic nodes in Panc02 tumor-bearing mice at 11 days after treatments indicated in (**a**). (*n* = 8 mice in each group for (**g**)). Scale bar: 10 mm for (**h**). **j**–**l** Numbers of CD8^+^Ki67^+^ T (**j**), CD8^+^IFN-γ^+^ T (**k**) and CD8^+^GzmB^+^ T cells (**l**) in tumor tissues of H22 tumor-bearing mice at 11 days after treatments indicated in **a** by flow cytometry. (*n* = 6 mice in each group). **m**, **n** Numbers of CD8^+^CD44^+^CD62L^+^ T (**m**) and CD8^+^CD44^+^CD62L^-^ T cells (**n**) in the blood of naïve mice, DOX@3D-MPs- or DOX@3D-MPs+anti-PD-1 antibody-cured H22 tumor-bearing mice indicated in **a** by flow cytometry. (*n* = 10 for naive mice, *n* = 3 and 9 for DOX@3D-MPs- and DOX@3D-MPs+anti-PD-1 antibody-cured mice, respectively). **o**, **p** Tumor volume curves of naive mice, DOX@3D-MPs- or DOX@3D-MPs+anti-PD-1 antibody-cured H22 tumor-bearing mice indicated in (**a**) after rechallenge with H22 (2 × 10^6^ cells) (**o**) and 4T1 (**p**) cells (5 × 10^5^ cells). (*n* = 10 for naive mice, *n* = 3 and 9 mice for DOX@3D-MPs- and DOX@3D-MPs+anti-PD-1 antibody-cured groups, respectively). **q**–**s** Percentages of Ki67^+^ (**q**), IFN-γ^+^ (**r**) and GzmB^+^ cells (**s**) in CD8^+^ T cells from H22 cell debris-restimulated splenocytes of naïve mice, DOX@3D-MPs- or DOX@3D-MPs+anti-PD-1 antibody-cured H22 tumor-bearing mice indicated in (**a**) by flow cytometry. (*n* = 10 for naive mice, *n* = 3 and 9 mice for DOX@3D-MPs- and DOX@3D-MPs+anti-PD-1 antibody-cured groups, respectively). **t** Cytotoxicity of T cells (effector cells) against H22 cells (target cells) at 24 h after T cells isolated from H22 cell debris-restimulated splenocytes of naive mice, DOX@3D-MPs- or DOX@3D-MPs+anti-PD-1 antibody-cured H22 tumor-bearing mice treated as indicated in (**a**) were incubated with H22 cells at different effector/target ratios by LDH assay. (*n* = 3). Data are shown as means ± s.d. *P < 0.05, **P < 0.01, ***P < 0.001

In addition to enhanced antitumor immunity and ameliorated tumor immunosuppressive microenvironment, DOX@3D-MPs treatment obviously upregulated the PD-1 expression of tumor-infiltrating CD8^+^ T lymphocytes (Supplementary Fig. 25a, c) and PD-L1 expression of tumor cells (Supplementary Fig. 25b, e) derived from H22 and 4T1 tumor-bearing mice. Moreover, a noticeable increase in the amounts of CD8^+^PD-1^+^CD69^+^ T lymphocytes in TIM was observed in mice treated by DOX@3D-MPs (Supplementary Fig. 25d). CD69 not only functioned as an activation marker showing that CD8^+^PD-1^+^ T lymphocytes still exhibited cytotoxic activity after DOX@3D-MPs treatment, recent works have shown that CD69 behaved as a potential factor predicting the response to PD-1/PD-L1 blockade therapy,^35,36^ which prompted us to treat tumor with DOX@3D-MPs in combination with anti-PD-1 antibody (denoted as DOX@3D-MPs+anti-PD-1) to further improve the anticancer effects. The antitumor capacity of the combination strategy was first evaluated in subcutaneous H22 tumor model. H22 tumor-bearing mice were administrated with PBS or DOX@3D-MPs via tail vein, followed with or without intravenous injection of anti-PD-1 antibody (Fig. 7a). As expected, anti-PD-1 antibody treatment alone elicited weak tumor inhibition compared with PBS (Fig. 7b, c and Supplementary Fig. 26a, b). DOX@3D-MPs noticeably hampered tumor growth (Fig. 7b), leading to 30% of mice being tumor free (Supplementary Fig. 26c). However, the treatment of DOX@3D-MPs+anti-PD-1 exhibited the most obvious tumor suppression effects, achieving 90% of mice being tumor free (Supplementary Fig. 26d) and 100% of mice surviving until 100 days after tumor inoculation (Fig. 7c). Moreover, the potent antitumor effects of DOX@3D-MPs+anti-PD-1 was additionally detected in mice bearing large established H22 tumor with the initial tumor sizes of 250 mm^3^, with 94.9% and 82.9% reduction in tumor volume (Supplementary Fig. 27a) and weight (Supplementary Fig. 27b), as well as much higher survival rate (Supplementary Fig. 27c). In addition, the synergistic curative effects of DOX@3D-MPs+anti-PD-1 was further validated by more malignant orthotopic 4T1 breast tumor (Fig. 7d, e) and orthotopic Panc02 pancreatic tumor models (Fig. 7f–i and Supplementary Fig. 28).

Consistently, the combined strategy of DOX@3D-MPs and anti-PD-1 antibody significantly elevated the amounts of CD8^+^Ki67^+^ T (Fig. 7j), CD8^+^IFN-γ^+^ T (Fig. 7k) and CD8^+^GzmB^+^ T cells (Fig. 7l) compared with other groups in large H22 tumors. Meanwhile, the numbers of Tcm and Tem (Supplementary Fig. 27d, e) cells were also obviously elevated in the spleens of large H22 tumor-inoculated mice treated by DOX@3D-MPs+anti-PD-1, indicating that the synergistic treatment of DOX@3D-MPs along with anti-PD-1 antibody evokes powerful immunogenic activation and immune memory for preventing tumor proliferation. To additionally estimate the long-term antitumor immune memory and its potential impact on preventing tumor recurrence, the amounts of Tcm and Tem cells were analyzed in the blood of the above treated H22 tumor-bearing mice with complete tumor ablation. Both the numbers of Tcm (Fig. 7m) and Tem cells (Fig. 7n) in the blood of three mice in DOX@3D-MPs- and nine mice in DOX@3D-MPs+anti-PD-1-treated groups with complete tumor ablation were markedly elevated in comparison with those of naïve mice. The strongest increase of the amounts of Tem cells (Fig. 7n) was detected in DOX@3D-MPs+anti-PD-1-treated group. Moreover, when these mice being completely cured were subjected to a second inoculation of H22 cells and a first injection of heterologous tumor cells like 4T1 cells at 95 days after initial treatment, all mice rejected H22 (Fig. 7o), but not 4T1 tumors (Fig. 7p) up to 28 days. However, 100% of H22 or 4T1 tumors continued to expand in untreated naïve mice subjected to subcutaneous inoculation of the identical amounts of H22 or 4T1 cells, respectively (Fig. 7o, p). These data indicate that the treatment of DOX@3D-MPs+anti-PD-1 might efficiently elicit antitumor immune memory to hinder tumor relapse. To further validate this, the splenocytes from the above mice with a second inoculation of tumor cells were subjected to restimulation with H22 cell lysates for three days, and the activation of CD8^+^ T cells and the cytotoxicity of these activated CD8^+^ T cells against tumor cells were assessed. Expectedly, the ratios of proliferated CD8^+^Ki67^+^ T cells (Fig. 7q), and cytotoxic CD8^+^IFN-γ^+^ T (Fig. 7r) and CD8^+^GzmB^+^ T cells (Fig. 7s) were noticeably increased in the splenocytes derived from mice treated by DOX@3D-MPs+anti-PD-1, much higher than those from naïve mice. Meanwhile, these restimulated T cells from the spleens of the mice received DOX@3D-MPs+anti-PD-1 treatment showed the strongest cytotoxicity against H22 cells (Fig. 7t), but not 4T1 cells (Supplementary Fig. 29). All above data reveal that the combination treatment strategy of DOX@3D-MPs along with anti-PD-1 antibody can elicit long-lasting tumor-specific immune memory against tumor recurrence.

**Fig. 8:**
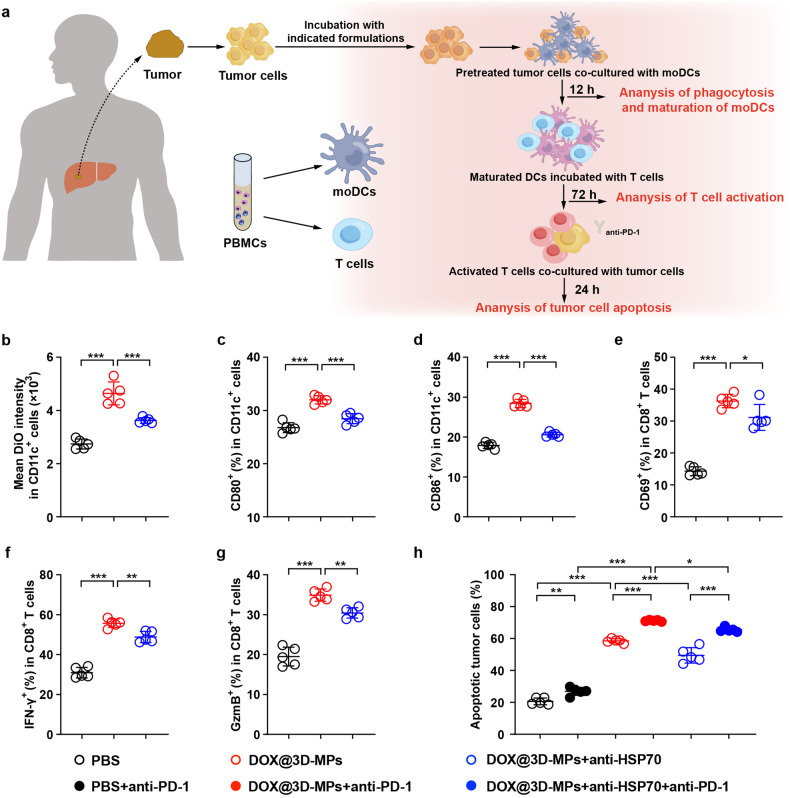
DOX@3D-MPs-induced antitumor immunity ex vivo. **a** Schematic illustration of ex vivo immune regulation analysis induced by DOX@3D-MPs. Tumor cells were isolated from tumor tissue of one liver cancer patient and then treated with PBS, DOX@3D-MPs or anti-HSP70 antibody pretreated-DOX@3D-MPs derived from HepG2 TRCs at DOX concentration of 1 μg mL^−1^ for 12 h. MoDCs and T cells were isolated from PBMCs derived from the same patient. moDCs were co-incubated with the above treated tumor cells for 12 h and then co-cultured with T cells for 3 days. The maturation of moDCs and activation of T cells were analyzed by flow cytometry. The above treated T cells were further co-incubated with tumor cells isolated from the same liver cancer patient in the presence or absence of anti-PD-1 antibody (20 μg mL^−1^) for 24 h, and the ratio of apoptotic tumor cells after T cells treatment was detected by flow cytometry. **b** Intracellular DiO MFI in CD11c^+^ moDCs after the immature moDCs were co-cultured with DiO-labeled tumor cells treated as indicated in (**a**) for 12 h. (*n* = 5). **c**, **d** Percentages of CD80^+^ (**c**) and CD86^+^ (**d**) cells in CD11c^+^ moDCs after the immature moDCs were co-cultured with tumor cells treated as indicated in **a** for 12 h by flow cytometry. (*n* = 5). **e**–**g** Percentages of CD69^+^ (**e**), IFN-γ^+^ (**f**) and GzmB^+^ (**g**) cells in CD8^+^ T cells after CD3^+^ T cells were incubated with the above matured moDCs as indicated in **c** for 3 days by flow cytometry. (*n* = 5). **h** Ratios of apoptotic tumor cells after the tumor cells were treated with the above activated T cells as indicated in **e** in the presence or absence of anti-human PD-1 antibody for 24 h. (*n* = 5). Data are shown as means ± s.d. *P < 0.05, **P < 0.01, ***P < 0.001

To further verify that the excellent anticancer capacity of the treatment of DOX@3D-MPs+anti-PD-1 was regulated by antitumor immunity, tumor cells isolated from fresh hepatocellular carcinoma (HCC) patient-derived tumor tissues were labeled with DiO and then treated with DOX@3D-MPs, followed by co-culture with monocyte-derived DCs (moDCs) from a healthy people’s peripheral blood mononuclear cells (PBMCs) (Fig. 8a). Consistently, moDCs phagocytosed more DOX@3D-MPs-treated tumor cells compared with PBS-treated group (Fig. 8b), leading to their efficient maturation as indicated by the elevated percentages of costimulatory molecules CD80^+^ (Fig. 8c) and CD86^+^ cells (Fig. 8d) in moDCs. Moreover, the ratios of CD69^+^ (Fig. 8e), INF-γ^+^ (Fig. 8f) and GzmB^+^ cells (Fig. 8g) in CD8^+^ T cells were obviously upregulated when T cells isolated from healthy PBMCs were incubated with the above moDCs matured by DOX@3D-MPs-treated tumor cells. Meanwhile, T cells from the above DOX@3D-MPs-treated group induced more apoptosis in tumor cells derived from the same HCC patient (Fig. 8h), confirming that the matured moDCs by DOX@3D-MPs-treated tumor cells effectively activated CD8^+^ T cells to exert cytotoxic effects against tumor cells. However, pretreatment with anti-HSP70 antibody significantly abrogated the phagocytosis of DOX@3D-MPs-treated tumor cells by moDCs (Fig. 8b) and the subsequent moDC maturation (Fig. 8c, d), as well as CD8^+^ T cell activation (Fig. 8e–g) and cytotoxicity against tumor cells (Fig. 8h), revealing that HSP70 in 3D-MPs was responsible for DOX@3D-MPs-induced immunogenic activation for tumor inhibition. Furthermore, combination of anti-PD-1 antibody and T cells from the above DOX@3D-MPs-treated group evoked the strongest apoptosis in tumor cells (Fig. 8h), confirming that the treatment of DOX@3D-MPs along with anti-PD-1 antibody elicited strong anticancer activity. On the whole, the above results demonstrate that DOX@3D-MPs effectively activate antitumor immunity to improve anti-PD-1 therapy in a HSP70-dependent manner.

## Discussion

Contemporary antitumor immunotherapy predominantly centers on T cell-mediated cellular immune responses,^37^ which is heavily reliant on the completeness of cancer-immunity cycle integrating a series of scenarios from the release of tumor-associated antigens, their phagocytosis and presentation by DCs, followed by tumor-specific T cell activation and culminating in the elimination of tumor cells.^38^ Chemotherapy, one of the most commonly used treatments, can elicit ICD and produce tumor antigens to induce antitumor immune responses.^39^ Unfortunately, chemoimmunotherapy faces limitations due to insufficient drug delivery and its focus on targeting only an aspect of the cancer-immunity cycle.^40^ Thus, efficiently delivering anticancer drugs to generate enough tumor antigens and simultaneously promoting antigen delivery to DCs and the subsequent antigen-presenting and T cell activation will help to improve antitumor immunity of cancer treatment.

In current study, we found that DOX@3D-MPs efficiently killed and induced ICD effects of tumor cells attributed to the high drug delivery efficiency of 3D-MPs, releasing more tumor antigens to initiate strong antitumor immune response. HSPs are a family of highly conserved intracellular chaperones that play crucial roles in a range of cellular processes to preserve cell integrity, maintain protein homeostasis and respond to stresses, including heat, hypoxia, oxidative stress or DNA damage.^41,42^ HSP70, the major stress-induced HSP, is expressed at low levels under normal conditions, while its expression is markedly increased in response to various extracellular stresses.^43^ Although HSP70 is localized intracellularly, it has been found to be translocated onto the cell membrane after stress and liberated into the extracellular environment in a membrane-dependent pattern,^44,45^ sharing the features of this protein in the cell membrane. HSP70 can mediate immune activation by capturing antigenic peptides and facilitating the transferring of the chaperoned cargoes to DCs for cross-presentation.^46,47^ Meanwhile, HSP70 also induces DC maturation and cytokine secretion, such as IL-1β, TNF-α and IL-6, functioning as an effective and safe immune adjuvant.^48,49^ In this work, we found that HSP70 was highly expressed in TRCs and DOX treatment further enhanced HSP70 expression in TRCs, resulting in the overexpression of HSP70 in DOX@3D-MPs. HSP70 in DOX@3D-MPs was located on the outer membrane of 3D-MPs, efficiently capturing tumor antigens released from DOX@3D-MPs-treated tumor cells and promoting their phagocytosis by DCs to evoke CD8^+^ T cell-dependent antitumor immunoregulation. The antitumor immunity mediated by CD8^+^ T cell was responsible for the potent anticancer activity of DOX@3D-MPs, as evidenced by the abrogation of DOX@3D-MPs-induced antitumor therapeutic efficacy in immunocompetent mouse models after CD8^+^ T cell depletion using anti-CD8 antibody and in the nude mouse tumor models.

The efficiency of anti-PD-1/PD-L1 therapy largely depends on the expression of PD-1/PD-L1 expression, the number of infiltrating T lymphocytes and the immunosuppressive microenvironment in tumor tissues.^50–52^ Several works have shown that T cells could be activated upon recognition of tumor antigens during which PD-1 was also upregulated via antigen-driven T cell receptor (TCR) signaling.^53–55^ Meanwhile, IFN-γ, as an essential cytokine released by the activated T cells, not only initiated anti-tumor immune response, but also upregulated the expression of PD-L1 in tumor cells to weaken the cytotoxic response for delicate balance between positive and negative immune signaling.^56,57^ Our work also demonstrated that DOX@3D-MPs efficiently elevated the amounts of CD8^+^ T cells and their activation (such as enhanced IFN-γ secretion) in tumor tissues, and simultaneously upregulated PD-1 expression in CD8^+^ T cells and PD-L1 expression in tumor cells of tumor tissues, successfully converting a “cold” tumor to a “hot” tumor. In addition, DOX@3D-MPs significantly decreased the amounts of Tregs and MDSCs, effectively improving tumor immunosuppressive microenvironment. Thus, DOX@3D-MPs efficiently boosted anti-PD-1 antibody therapy in multiple tumor models, including large subcutaneous H22 hepatoma tumor model, orthotopic 4T1 breast tumor and Panc02 pancreatic tumor models, and even in HCC patient-derived tumor tissues, generating strong antitumor immune memory to inhibit tumor recurrence. In view of the similar mechanisms, whether DOX@3D-MPs improve other immunotherapies like anti-CTLA4 and CAR-T therapy in solid tumors remains to be explored. In addition, single-cell RNA sequencing (scRNA-seq) should be performed to define the accurate immune cell types in order to better clarify the mechanisms on the potent anticancer effects of DOX@3D-MPs in the future research.

In spite of the promising outcomes and favorable safety of the treatment of DOX@3D-MPs along with anti-PD-1 antibody, there are still many issues to be taken care for this treatment platform from bench to human clinical translation. Firstly, while our assessment of DOX@3D-MPs’ antitumor effect and immune response involved subcutaneous and orthotopic tumor models, as well as ex vivo clinical samples, these models might not reflect the complex tumor microenvironment of primary tumors. Therefore, the establishment of additional primary tumor models, including those that better mimic the intricacies of human tumors, such as patient-derived xenografts (PDX) tumor models, is imperative to provide a more accurate estimation of DOX@3D-MPs’ antitumor efficacy. Secondly, our evaluation of the immune response heavily relied on single-cell flow cytometric analysis, potentially limiting to capture the spatial information and intricate interactions occurring within the intricate tumor microenvironment. Thus, the development of more comprehensive immunophenotyping and spatial analysis of their interactions is warranted. Thirdly, the cell source for DOX@3D-MPs production would be a crucial issue. Using 3D-MPs derived from patient-derived TRCs as anticancer drug carriers is a potential direction for personalized treatment aiming at avoiding the attack by immune systems. Considering the convenience and feasibility in fabrication, TRCs derived from immortalized human tumor cells are a good replacement as the donor cells. However, the potential security risk would need a careful and systemic assessment. In addition, appropriate methods for scaled-up production of DOX@3D-MPs are urgently necessary. Lastly, besides DOX, more drug candidates, such as microRNA (miRNA) which can regulate immune checkpoint pathways, tumor immune evasion and tumor microenvironment characteristics, and the proper clinical indications are needed to be considered for achieving better tumor therapeutic efficacy.

In summary, we demonstrate that DOX@3D-MPs efficiently induce ICD of tumor cells, resulting in the release of sufficient tumor antigens. HSP70 expressed on 3D-MPs captures the released tumor antigen and assists DCs to phagocytose more tumor antigens, triggering the efficient antigen-presenting function of DCs and activation of CD8^+^ T cells. DOX@3D-MPs significantly potentiate the effectiveness of anti-PD1 antibody therapy against cancer and induce long-lasting immune memory to hinder tumor recurrence. These findings demonstrate that DOX@3D-MPs serve as a promising agent to boost the therapeutic benefits of anti-PD-1 therapy.

## Materials and methods

### Materials

Dulbecco’s Modified Eagle’s Medium (DMEM), RPMI 1640 medium, fetal bovine serum (FBS), penicillin, streptomycin and collagenase type I were obtained from Gibco BRL/Life Technologies (Grand Island, NY, USA). Doxorubicin hydrochloride (DOX.HCl) was purchased from Beijing HuaFeng United Technology CO., Ltd. (Beijing, China). Doxil was provided from Fudan-Zhangjiang Bio-Pharmaceutical (Shanghai, China). Thrombin and fibrinogen were obtained from Searun Holdings Company (Freeport, ME, USA). Mouse granulocyte-macrophage colony- stimulating factor (GM-CSF), IL-4 and IL-2 were sourced from PeproTech (Rocky Hill, NJ, USA). Human GM-CSF, IL-2, IL-4 and antibodies applied for flow cytometry analysis of immune cells both from mice and human were obtained from Biolegend (San Diego, CA, USA).

### Cell culture

H22, 4T1, Panc02 and HepG2 cells were acquired from Type Culture Collection of the Chinese Academy of Sciences (Shanghai, China). B16 and B16-OVA cells were generously provided by Prof. Bo Huang (Institute of Basic Medical Sciences, Chinese Academy of Medical Sciences, Beijing, China). H22, 4T1 and B16-OVA cells were maintained in RPMI 1640 medium, while Panc02 and HepG2 cells were cultured in DMEM medium supplemented with 10% FBS, 100 μg mL^−1^ streptomycin and 100 U mL^−1^ penicillin at 37 °C in a 5% CO_2_ incubator. TRCs were obtained by using soft 3D fibrin gels with softness of 90 Pa as described.^22^ Briefly, single-cell suspensions (1 × 10^4^ cells mL^−1^) of differentiated tumor cells (including H22, 4T1, B16, Panc02 or HepG2 cells) were added at the volume ratio of 1:1 with the fibrinogen prediluted by T7 solution (0.15 M NaCl and 0.05 M Tris at pH 7.4) at the concentration of 2 mg mL^−1^. Then 0.25 mL fibrinogen/cell mixtures were added to individual wells of a 24-well plate which was pretreated with 5 µL pre-cooled thrombin at a concentration of 0.1 U µL^−1^. The plate was then placed in a 37 °C incubator with 5% CO_2_ for 30 min. Afterwards, 1 mL complete DMEM medium was added to each well of the plate. On the fifth day of incubation, the cell spheroids in 3D gels were collected and digested with 0.08% (w/v) collagenase and 0.4% (w/v) dispase II at 37 °C for 15 min to acquire single cells. Then the collected cells were maintained in serum-free DMEM/F-12 medium containing 20 ng mL^−1^ mouse epidermal growth factor (mEGF), 2% B27 and 1% glutamine, and passaged for about 20 generations to obtain large-scale TRCs for subsequent experiments.

Murine BMDCs were isolated from femurs and tibias of 8-week-old male BALB/c mice and cultured in RPMI 1640 containing 10% FBS, 100 U mL^−1^ penicillin, 100 μg mL^−1^ streptomycin, 10 ng mL^−1^ murine GM-CSF and 10 ng mL^−1^ murine IL-4. Murine CD3^+^ T cells were dissociated from the spleens of male BALB/c mice (8-week-old) by utilizing a MokoSort^TM^ Mouse CD3^+^ T cell isolation kit (Biolegend, USA) and magnified for 5 days in RPMI 1640 medium supplemented with 10% FBS, 100 μg mL^−1^ streptomycin, 100 U mL^−1^ penicillin and 20 ng mL^−1^ murine IL-2. To isolate human PBMCs, healthy human blood was collected and subjected Ficoll gradient for isolation of PBMCs. The isolated PBMCs were cultured in RPMI 1640 containing 10% FBS, 100 U mL^−1^ penicillin, 100 μg mL^−1^ streptomycin, 10 ng mL^−1^ human GM-CSF and 10 ng mL^−1^ human IL-4 for 7 days for the induction of moDCs. Human CD3^+^ T cells were isolated from PMBCs by using a MokoSort^TM^ Human CD3^+^ T cell isolation kit (Biolegend, USA) and amplified for 5 days in RPMI 1640 medium supplemented with 10% FBS, 100 μg mL^−1^ streptomycin, 100 U mL^−1^ penicillin and 20 ng mL^−1^ human IL-2.

### Preparation of DOX@3D-MPs

TRCs (including H22 TRCs, 4T1 TRCs, B16 TRCs, Panc02 TRCs and HepG2 TRCs) were collected and exposed to ultraviolet irradiation (UVB) at a dose of 300 J m^−2^ for 60 min, then the cells were incubated with DOX at the concentration of 200 μg mL^−1^ for 16 h. The collected supernatants were subjected to centrifugation at 600 × *g* for 10 min to remove the cells, and then centrifuged at 14,000 *g* for 2 min to remove the large cell debris. The supernatants were subsequently subjected to ultimate centrifugation at 14,000 *g* for 1 h to acquire DOX@3D-MPs. The pellets were rinsed with PBS for three times and resuspended in PBS for the subsequent analysis. The empty 3D-MPs were obtained though the same procedure without incubation with DOX.

### Animal models

C57BL/6 mice (male, 6–8 weeks old) and BALB/c mice (male and female, 6–8 weeks old) were procured from Beijing Vital River Laboratory Animal Technology Co., Ltd. (Beijing, China). These mice were accommodated in an animal facility under proper administration. To establish H22 tumor model, male BALB/c mice were subjected to subcutaneous inoculation of H22 cells at a concentration of 2 × 10^6^ cells into the flanks. To create a tumor model to stimulate the tumor recurrence, H22 tumor-bearing mice that had been previously cured by treatment with DOX@3D-MPs or DOX@3D-MPs+anti-PD-1 were subcutaneously inoculated with 2 × 10^6^ H22 cells in the left flank and 5 × 10^5^ 4T1 cells in the right flank. Additionally, orthotopic 4T1 tumor-inoculated mice were established by injecting 5 × 10^5^ 4T1 cells into the right fourth breast fat pad of female BALB/c mice. To establish the lung metastasis model, 1 × 10^5^ 4T1-Luc cells were inoculated via tail vein. The orthotopic Panc02 tumor model was established by inoculating Panc02 cells into pancreatic tails of C57BL/6 mice. All animal experiments conducted in current study were performed in accordance with the established guidelines and were approved by the Institutional Animal Care and Use Committee at Tongji Medical College, Huazhong University of Science and Technology (Wuhan, China).

### In vivo antitumor effects

H22, 4T1 or Panc02 tumor-inoculated mice were administrated with six doses of PBS, 3D-MPs, free DOX, 3D-MPs+DOX or DOX@3D-MPs by two days interval at the DOX dose of 0.5 mg kg^−1^, or three doses of Doxil at 4 mg kg^−1^ by three days interval via tail vein, with or without three doses of anti-PD-1 antibody at the dosage of 5 mg kg^−1^ by three days interval administrated intraperitoneally. The tumor sizes of H22- or 4T1-tumor bearing mice were assessed daily using a Vernier Caliper and the body weights of the mice were recorded. For antitumor effect analysis, a portion of the randomly selected mice was subjected to euthanization, and both tumor tissues and essential organs (including heart, liver, spleen, lung and kidney) were collected. The tumor weights were measured. The collected main organs were preserved by fixation with 4% paraformaldehyde, followed by sectioning and examination through H&E staining to assess any structural or histological changes. The remaining mice were utilized for the observation of long-range tumor hindrance and survival ratio. For analysis of lung metastasis in 4T1 metastatic tumor model, the lungs were fixed in Bouin’s solution (Solarbio, Beijing, China) over 12 h, and the metastatic tumor nodes present in the lungs were counted, fixed, and sectioned for subsequent H&E staining to examine their histological characteristics. For in vivo observation of lung metastasis and tumor recurrence, the tumor-bearing mice were intraperitoneally injected with D-luciferin potassium salt at dosage of 150 mg kg^−1^ and then imaged though the Caliper IVIS Lumina II 701 bioluminescence imaging system (PerkinElmer, Waltham, MA, USA) after a 5 min interval every 4 days.

### Cell viability assay

H22, 4T1 or B16-OVA cells were subjected to incubation with DOX, 3D-MPs+DOX or DOX@3D-MPs for 24 h, in which the applied drug concentration is 1 μg mL^−1^ of DOX. The cell viability was determined by utilizing cell counting kit-8 (CCK-8) assay. Briefly, at the end of treatment, the cells were co-incubated with CCK-8 solution (10 μL, Dojindo, Japan) for 4 h incubation period. Subsequently, the absorption value at wavelength 450 nm was determined using a MultiSkan FC microplate reader (Thermo Scientific, USA).

### In vitro ICD detection, BMDC maturation, T cell activation and cytotoxicity against tumor cells

H22, 4T1 or B16-OVA cells were subjected to incubation with PBS, 3D-MPs, DOX, 3D-MPs+DOX or DOX@3D-MPs for 12 h, in which the applied drug concentration is 1 μg mL^−1^ of DOX. The supernatants were collected. The HMGB1 and ATP in the supernatants were detected though the HMGB1 ELISA kit (Moshake, China) and enhanced ATP assay kit (Beyotime, China), respectively. The collected cells were treated with mouse CRT-specific antibody (catalog #MAB 38981, clone 681233, R&D Systems, USA) for 30 min, followed by subjecting to goat anti-mouse antibody conjugated with FITC (Beyotime, catalog #A0568, diluted to 1/100) for 60 min. The CRT^+^ cell ratio was measured by the CytoFLEX S flow cytometry (Beckman Coulter, Fullerton, CA, USA).

The above treated H22, 4T1 or B16-OVA cells were stained with DiO and incubated with immature BMDCs for 12 h. BMDCs were then marked with fluorescence-conjugated anti-CD11c (catalog #117328, clone No418, diluted to 1/80), CD80 (catalog #104725, clone 16–10A1, diluted to 1/20), CD86 (catalog #105012, clone GL-1, diluted to 1/80) or H-2Kb-OVA(catalog #141605, clone 25-D1.16, diluted to 1/20). The phagocytosis of tumor antigens by BMDCs (CD11c^+^DiO^+^ cells) and the maturation of BMDCs were analyzed by flow cytometry. To further evaluate T lymphocyte activation, the BMDCs that have undergone above treatment were co-cultured for three days with murine CD3^+^ T cells at the BMDC/T cell ratio of 1:4. The CD3^+^ T cells were obtained and stimulated with cell activation cocktail (Biolegend, catalog #423304) containing phorbol 12-myristate-13-acetate (PMA, 40.5 µM), ionomycin (669.3 µM), and protein transport inhibitor Brefeldin A (2.5 mg mL^−1^) for 3 h. The activated cells were collected and incubated with fluorescence-labeled anti-mouse CD3 (catalog #100228, clone 17A2, diluted to 1/20) and CD8 (catalog #100722, clone 53–6.7, diluted to 1/40) antibody for surface staining. To analyze the intracellular cytokines, the cells were fixed with fixation buffer (Biolegend, catalog #420801) and subsequently re-suspended in permeabilization wash buffer (Biolegend, catalog # 421002). The cells were subjected to re-staining using fluorescence-conjugated anti-IL-2 (catalog #503808, clone JES6–5H4, diluted to 1/20), IFN-γ (catalog #505810, clone XMG1.2, diluted to 1/20), TNF-α (catalog #506305, clone MP6-XT22, diluted to 1/80) or Granzyme B (catalog #372204, clone QA16A02, diluted to 1/20) antibody for ultimate flow cytometric analysis after get rid of the unbounded antibody by rinsing with PBS. To further evaluate the cytotoxicity of activated T cell against tumor cells, the T cells were harvested and co-cultured with tumor cells at different ratio of T cells/tumor cells. After 24 h co-culture, the lactate dehydrogenase (LDH) release of tumor cells was detected by using a cytotoxicity LDH assay kit (Dojindo, Kumamoto, Kyushu, Japan) following the manufacturer’s instructions.

### TIM analysis

After treatment, the tumors, spleens and TDLNs of H22 tumor-bearing mice were obtained. The tumor masses were split and then treated with cell culture medium with 5 μg mL^−1^ DNase I and 0.8 mg mL^−1^ collagenase type I for 1 h at 37 °C. The tumor homogenates were centrifuged at 350 × *g* for 5 min, rinsed with PBS, and treated with red blood cell (RBC) lysing buffer (Biosharp, Hefei, China) to lyse the RBCs. The cells were suspended in PBS and filtered twice with a 40 μm nylon mesh to acquire single-cell suspensions. The single-cell suspensions were then added upon the density gradient media Ficoll-Paque PLUS (GE Healthcare, Piscataway, NJ, USA) and centrifuged to isolate the tumor-infiltrating lymphocytes. The lymphocytes from spleens and TDLNs were collected by squashing the tissues to pass through a 40 μm leach and then get rid of the RBCs. The isolated cells were treated following the manufacturer’s instructions with different fluorescent antibodies, including anti-CD11c (catalog #117328, clone N418, diluted to 1/80), CD80 (catalog #104708, clone 16–10A1, diluted to 1/40), CD86 (catalog #105012, clone GL-1, diluted to 1/80), MHC-II (catalog #107620, clone M5/114.15.2, diluted to 1/200), CD45 (catalog # 103132, clone 30-F11, diluted to 1/50; catalog #103112, clone 30-F12, diluted to 1/100), CD3 (catalog #100228, clone 17A2, diluted to 1/20; catalog #100320, clone145–2C11, diluted to 1/50), CD8 (catalog # 100722, clone 53–6.7, diluted to 1/40; catalog #100706, clone 53–6.7, diluted to 1/80), CD4 (catalog #130308, clone H129.29, diluted to 1/200), CD69 (catalog #104513, clone H1.2F3, diluted to 1/20), CD279 (PD-1) (catalog #135221, clone 29F.1A12, diluted to 1/200), CD25 (catalog #102012, clone PC61, diluted to 1/80), CD11b (catalog #101206, clone M1/70, diluted to 1/200) and Ly-6G/Ly-6C (Gr-1) (catalog #108408, clone RB6–8C5, diluted to 1/80). For detection of secreted cytokines, the cells were pretreated with cell activation cocktail (catalog #423304, diluted to 1/500) containing PMA (40.5 µM), ionomycin (669.3 µM) and protein transport inhibitor Brefeldin A (2.5 mg mL^−1^). Then the cells were fixed and permeabilized with fixation buffer and permeabilization wash buffer, respectively and further stained with fluorescence-labeled anti-IL-2, IFN-γ, TNF-α or Granzyme B antibody. To stain the transcriptional factors, the above surface-stained cells were subjected to a commercial buffer (Biolegend, catalog #424401). Subsequently, the cells were stained with fluorescence-conjugated anti-FoxP3 (catalog #126419, clone MF-14, diluted to 1/40). To stain Ki67, the surfaced-stained cells were subjected to 70% ethanol precooled to −20 °C for 1 h and stained with fluorescence-conjugated anti-Ki67 (catalog #652410, clone MF-14, diluted to 1/40). Tcm and Tem from spleens were determined by staining with fluorescence-conjugated anti-CD3 (catalog #100228, clone 17A2, diluted to 1/20), CD8 (catalog #10722, clone 53–6.7, diluted to 1/40), CD44 (catalog #103005, clone IM7, diluted to 1/200) and CD62L (catalog #104411, clone MEL-14, diluted to 1/80). All antibodies were incubated with the cells at indicated dilution for 30 min in the dark at room temperature. The cells were subsequently subjected to flow cytometric analysis by the CytoFLEX S (Beckman, USA). Gating strategies for flow cytometry analysis of cell types involved in this study have been presented in Supplementary Fig. 30.

### In vitro T cell function analysis

The splenocytes were isolated from the spleens of 4T1 tumor-bearing mice after treatments and the T cell function was then determined by IFN-γ ELISpot analysis using a mouse IFN-γ precoated ELISpot kit (Dakewe Biotech Co., Ltd, Beijing, China). Briefly, the isolated splenocytes (1 × 10^5^ cells per well) were seeded in a 96-well plate pre-coated with mouse anti-IFN-γ antibody on PVDF membrane and then incubated with tumor cell lysates (1 μg mL^−1^) for 24 h according to the manufacturer’s instruction. The secreted IFN-γ was captured by IFN-γ-specific biotinylated antibody and formed as brown spots on the PVDF membrane. The spots were quantified and pictured by Dakewe Biotech Co., Ltd. For cytotoxic assay, the isolated splenocytes were rechallenged with 4T1 cell lysates (1 μg mL^−1^) for 3 days. The re-stimulated splenocytes were harvested and co-cultured with 4T1 cells at different ratios of splenocytes/tumor cells. After 24 h co-culture, the LDH release of 4T1 cells was detected by using a cytotoxicity LDH assay kit in accordance to the manufacturer’s directions.

### Stable knockdown of HSP70 in H22 TRC cells

For construction of the stable knockdown of HSP70 in H22 TRCs, H22 TRCs were transfected with lentivirus carrying CRISPR/Cas9 elements. The lentivirus was packaged in 293T cells with DNA vector lentiCRISPR v2 (Addgene plasmid 52961), envelope plasmid pMD2.G (Addgene plasmid 12259) and packaging plasmid psPAX2 (Addgene plasmid 12260) at the ratio of 4:1:3. To target HSP70, two guide sequences, including sgRNA1: CACCGCAGCGCCACCTGGTTCTTGG (sense) and AAACCCAAGAACCAGGTGGCGCTGC (antisense), sgRNA2: CACCGCAGGTGCGTGTCGCCCGCCG (sense) and AAACCGGCGGGCGACACGCACCTGC (antisense), were cloned into the DNA vector. After transfection, H22 TRC cells were subjected to selection with 1 μg mL^−1^ puromycin for 1 week and maintained in a culture medium containing 0.5 μg mL^−1^ puromycin. The knockdown of HSP70 in H22 TRCs was tested by western blot analysis.

### Immune TEM analysis

DOX@3D-MPs were incubated with 5 μg mL^−1^ HSP70 antibody (Proteintech, catalog #10995-1-AP, China) at 4 °C for 1 h and rinsed with PBS twice to get rid of the unbound antibody. DOX@3D-MPs were then incubated with secondary antibody of goat anti-rabbit IgG conjugated with 20 nm gold nanoparticles (Abcam, catalog #ab270563, diluted to 1/20) at room temperature for 1 h. The HSP70 on DOX@3D-MPs was examined by TEM (HT7700, Hitachi, Japan) with an 80 kV accelerating voltage.

### Antigen capture by 3D-MPs and phagocytosis by DCs

To estimate the antigen capture ability of 3D-MPs, FITC-labeled OVA protein or DiO-labeled H22 tumor cell lysates (1 μg mL^−1^) were incubated with 3D-MPs, 3D-MPs_EV_ or 3D-MPs_HSP70-KO_ (10 μg protein mL^−1^) at 37 °C for 1 h and rinsed with PBS. The FITC or DIO fluorescence intensity in 3D-MPs was then estimated by CytoFLEX S flow cytometry. To further estimate the 3D-MPs-mediated antigen phagocytosis by DCs, BMDCs were incubated with 3D-MPs, 3D-MPs_EV_ or 3D-MPs_HSP70-KO_ (10 μg protein mL^−1^) in the presence of FITC-labeled OVA protein or DiO-labeled H22 cell lysates (1 μg mL^−1^) for 12 h. BMDCs were then incubated with fluorescence-labeled anti-CD11c (catalog #117328, clone No418, diluted to 1/80), and the phagocytosis of OVA or H22 cell lysates by DCs (CD11c^+^FITC^+^ or CD11c^+^DiO^+^ cells) were determined by flow cytometry.

### Ex vivo antitumor immunity of DOX@3D-MPs

Patient-derived HCC tumor tissue was collected from one patient undergoing routine surgery at the Tongji Hospital, Tongji Medical College of Huazhong University of Science and Technology (Wuhan, China). Ethical permission for this study was obtained from the Clinical Trail Ethics Committee of Huazhong University of Science and Technology, and the patient provided informed consent to this study.

The tumor tissue was cut into small pieces and digested in RPMI 1640 medium containing 0.8 mg mL^−1^ type I collagenase and 0.25 mg mL^−1^ trypsin at 37 °C for 20 min and mixed gently with pipette every 5 min. The homogenates were filtered twice through a 40 μm cell strainer and washed twice with PBS. The isolated tumor cells were subsequently maintained in RPMI 1640 medium supplemented with 20% FBS, 100 U mL^−1^ penicillin and 100 μg mL^−1^ streptomycin. The tumor cells were treated with PBS, DOX@3D-MPs or anti-HSP70 antibody-pretreated DOX@3D-MPs derived from HepG2 TRCs at DOX concentration of 1 μg mL^−1^ for 12 h. The tumor cells were stained with DiO and incubated with immature moDCs derived from a healthy donor for 12 h. MoDCs were then stained with fluorescence-labeled anti-CD11c (catalog #301613, clone 3.9, diluted to 1/20), CD80 (catalog #375407, clone W171490, diluted to 1/20), or CD86 (catalog #374203, clone BU63, diluted to 1/20). The phagocytosis of tumor antigens by moDCs (CD11c^+^DiO^+^ cells) and the maturation of moDCs were analyzed through flow cytometry. To further evaluate T lymphocyte activation, moDCs that have undergone the above treatment were co-incubated with human CD3^+^ T cells derived from a healthy donor at the moDC/T cell ratio of 1:4 for three days. The CD3^+^ T cells were harvested and treated with cell activation cocktail with Brefeldin A for 3 h. The activated cells were collected and incubated with fluorescence-labeled anti-human CD3 (catalog #317333, clone OKT3, diluted to 1/20) and CD8 (catalog #344717, clone SK1, diluted to 1/20) antibody for surface staining. After fixation and permeabilizationhe cells using fixation buffer and permeabilization wash buffer, respectively, the cells were further stained with fluorescence-labeled CD69 (catalog #310909, clone FN50, diluted to 1/20), IFN-γ (catalog #502511, clone 4S.B3, diluted to 1/20) or Granzyme B (catalog #396409 clone QA18A28, diluted to 1/20). After staining, the cells were analyzed by CytoFLEX S flow cytometry. To further evaluate the cytotoxicity of activated T cell against HCC tumor cells, the above treated T cells were harvested and co-cultured with tumor cells derived from the same patient at different T cell/tumor cell ratios with or without anti-PD-1 antibody (20 μg mL^−1^). After 24 h co-culture, tumor cells were first gated by their negative expression of the human CD45 marker using an anti-human CD45 antibody (catalog #304005, clone HI30, diluted to 1/20). The isolated tumor cells were stained with anti-Annexin V (catalog #640920) and Zombie Violet (catalog #423113) for apoptosis analysis. All antibodies applied for flow cytometry analysis were purchased from Biolegend (San Diego, CA, USA) and incubated with the cells according to the instructions.

### Statistics analysis

All experiments were repeated at least three times independently. All data are presented as means ± s.d. and analyzed by one-way ANOVA or two-way ANOVA for comparison of multiple groups. Statistical analysis was conducted using the GraphPad Prism 9 software. *P* values < 0.05 was considered statistically significant.

### Supplementary information

Supplemental Material

## Data Availability

The data that support the findings of this study are available within the paper and its Supplementary Information files. Raw data are available upon reasonable request from the corresponding author.
