# Supplementary material for: Tumor-repopulating cell-derived microparticles elicit cascade amplification of chemotherapy-induced antitumor immunity to boost anti-PD-1 therapy
Source: Signal Transduct Target Ther. 2023 Oct 25;8:408. doi: 10.1038/s41392-023-01658-3 (PMC10598206; doi:10.1038/s41392-023-01658-3)
Supplement: Supplementary file 1 — Supplemental Material [file 41392_2023_1658_MOESM1_ESM.docx]

Supplementary Materials for

Tumor-repopulating cell-derived microparticles enhance chemotherapy-induced antitumor immunity and boosting anti-PD-1 therapy

Nana Bie^1,#^, Tuying Yong^1,2,3,#^, Zhaohan Wei^1,#^, Qingle Liang^1^, Xiaoqiong Zhang^1^, Shiyu Li^1^, Xin Li^1^, Jianye Li^1^, Lu Gan^1,2,3,^*, Xiangliang Yang^1,2,3,^*

*Correspondence to: lugan@mail.hust.edu.cn; yangxl@mail.hust.edu.cn.

**This PDF file includes:**

Supplementary Fig. 1-30 with their legends


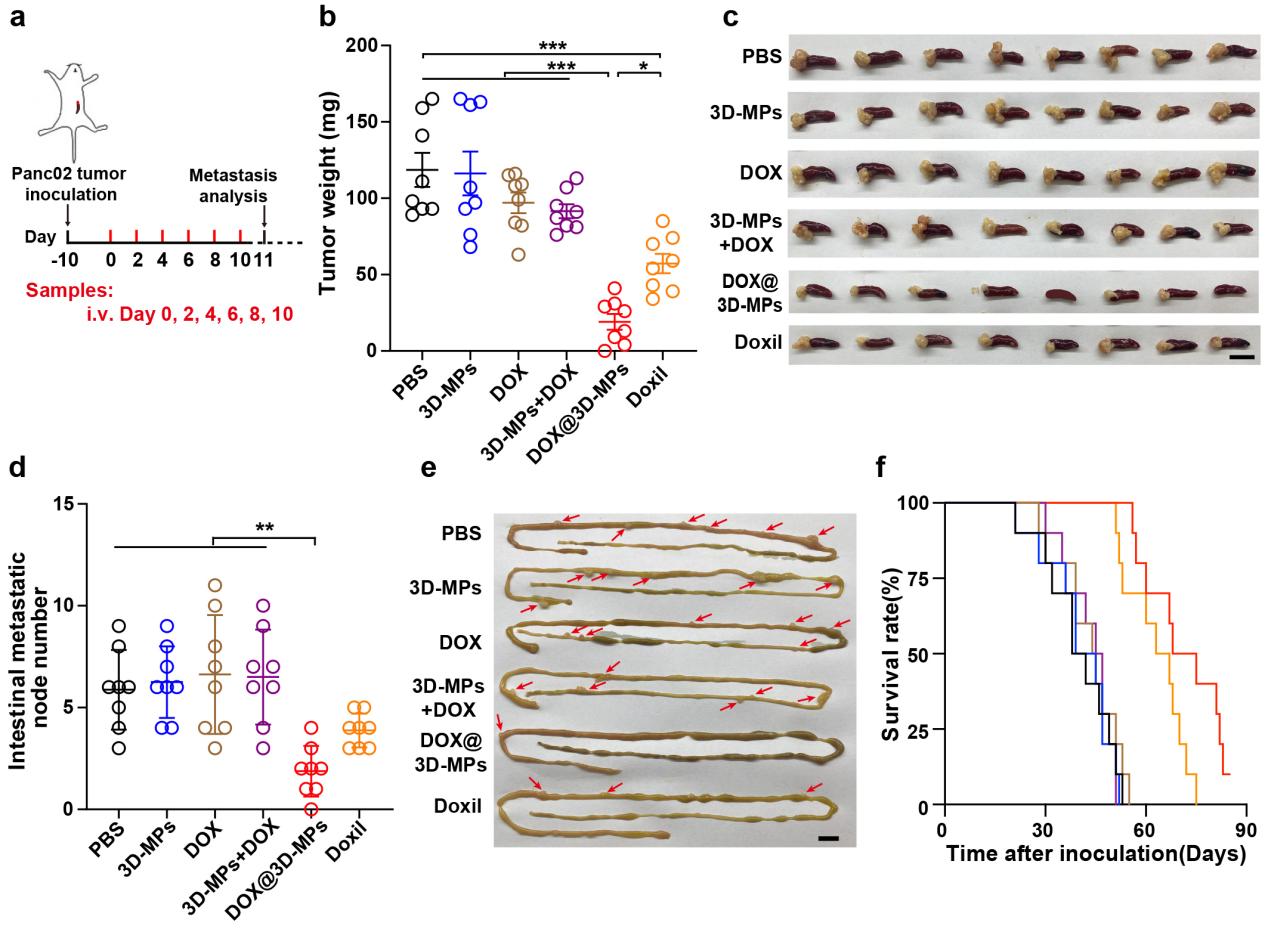


**Supplementary Fig. 1** **Potent antitumor activity of DOX@3D-MPs in orthotopic Panc02 tumor-bearing mice.** **a** Schematic schedule for the antitumor experiments in orthotopic Panc02 tumor-bearing mice after intravenous injection of PBS, 3D-MPs, DOX, 3D-MPs+DOX or DOX@3D-MPs derived from Pan02 TRCs at DOX dosage of 0.75 mg kg^−1^ once every other day for 6 times, or high dosage of Doxil at 4 mg kg^−1^ once every three days for 3 times. **b, c** Tumor weights (**b**) and images of pancreatic tumors (**c**) of orthotopic Panc02 tumor-bearing mice at 11 days after treatments indicated in **a.** Data are presented as means ± s.d. for **b** (n = 8 mice per group). Scale bar: 10 mm for **c**. **d, e** Numbers (**d**) and representative images (**e**) of intestine metastatic nodes in Panc02 tumor-bearing mice at 11 days after treatments indicated in **a**. Data are presented as means ± s.d. for **d** (n = 8 mice per group). Scale bar: 10 mm for **e**. **f** Kaplan-Meier survival plots of Panc02 tumor-bearing mice after treatments indicated in **a** (n = 10 mice per group). **P* < 0.05, ***P* < 0.01, ****P* < 0.001.

**
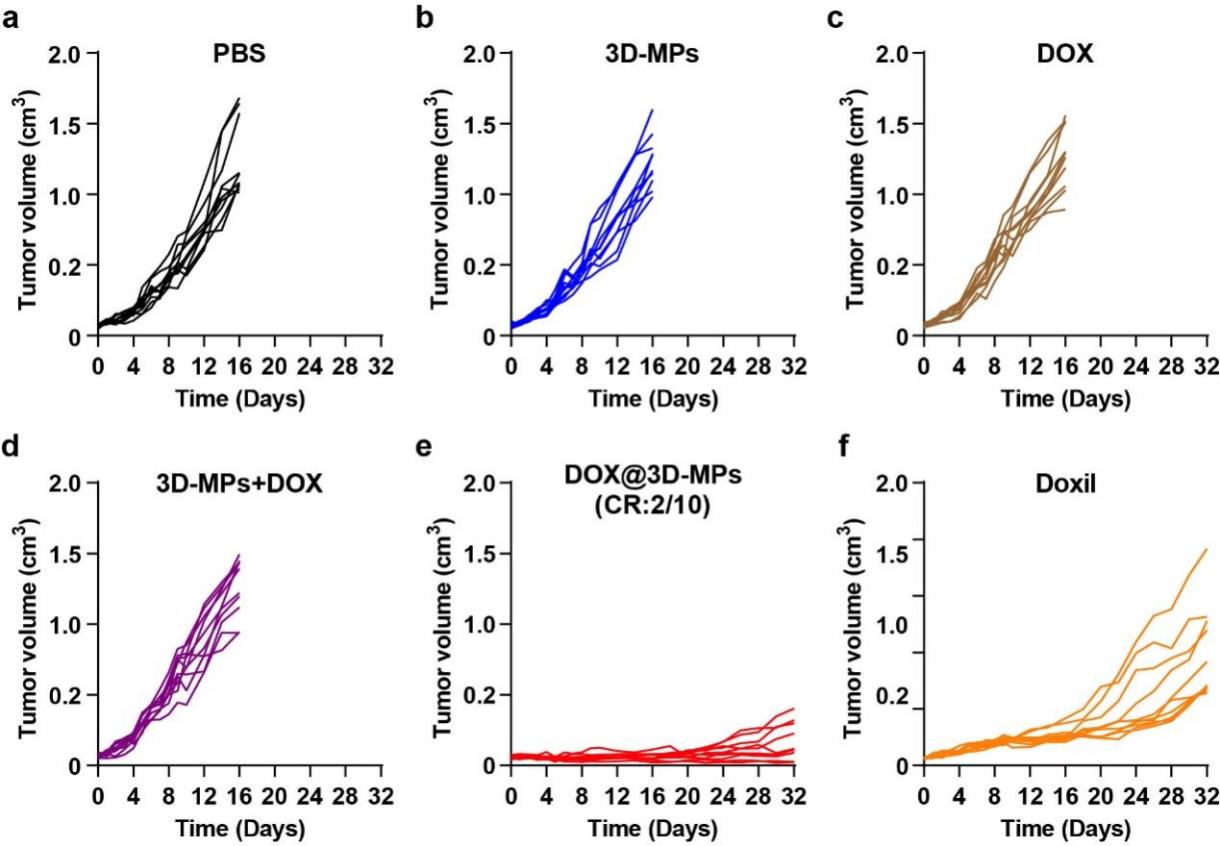
**

**Supplementary Fig. 2** **Potent antitumor activity of DOX@3D-MPs in subcutaneous H22 tumor-bearing mice. a-f** Individual tumor growth curves of H22 tumor-bearing mice after intravenous injection of PBS (**a**), 3D-MPs (**b**), DOX (**c**), 3D-MPs+DOX (**d**), DOX@3D-MPs (**e**) derived from H22 TRCs at DOX dosage of 0.75 mg kg^−1^ once every other day for 6 times, or high dosage of Doxil (**f**) at 4 mg kg^−1^ once every three days for 3 times (n = 10 mice per group). CR: complete response.

**
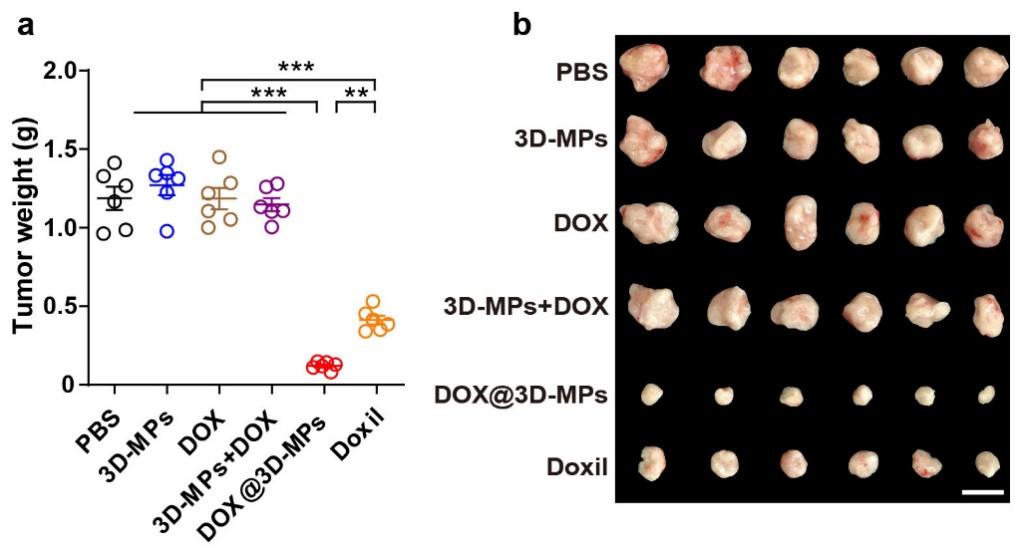
**

**Supplementary Fig. 3** **Potent antitumor activity of DOX@3D-MPs in subcutaneous H22 tumor-bearing mice. a, b** Tumor weights (**a**) and images of tumor tissues (**b**) of H22 tumor-bearing mice at 11 days after intravenous injection of PBS, 3D-MPs, DOX, 3D-MPs+DOX, DOX@3D-MPs derived from H22 TRCs at DOX dosage of 0.75 mg kg^−1^ once every other day for 6 times, or high dosage of Doxil at 4 mg kg^−1^ once every three days for 3 times. Data are presented as means ± s.d. for **a** (n = 6 mice per group) . ***P* < 0.01, ****P* < 0.001. Scale bar: 10 mm for **b**.


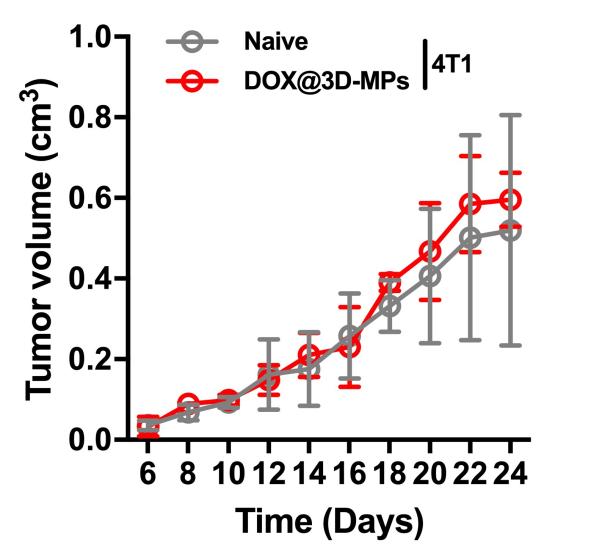


**Supplementary Fig. 4** Tumor growth curves of naive mice or DOX@3D-MPs-cured mice indicated in Fig. **2a** after rechallenge with 4T1 cells (5 × 10^5^ cells). Data are presented as means ± s.d. (n = 10 for naïve mice, n = 2 for DOX@3D-MPs-cured mice).


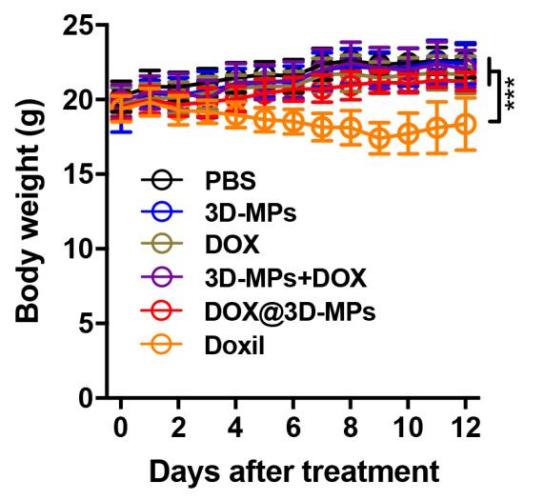


**Supplementary Fig. 5** Body weights of subcutaneous H22 tumor-bearing mice after intravenous injection of PBS, 3D-MPs, DOX, 3D-MPs+DOX, DOX@3D-MPs derived from H22 TRCs at DOX dosage of 0.75 mg kg^−1^ once every other day for 6 times, or high dosage of Doxil at 4 mg kg^−1^ once every three days for 3 times. Data are presented as means ± s.d. (n = 10 mice per group). ****P* < 0.001.


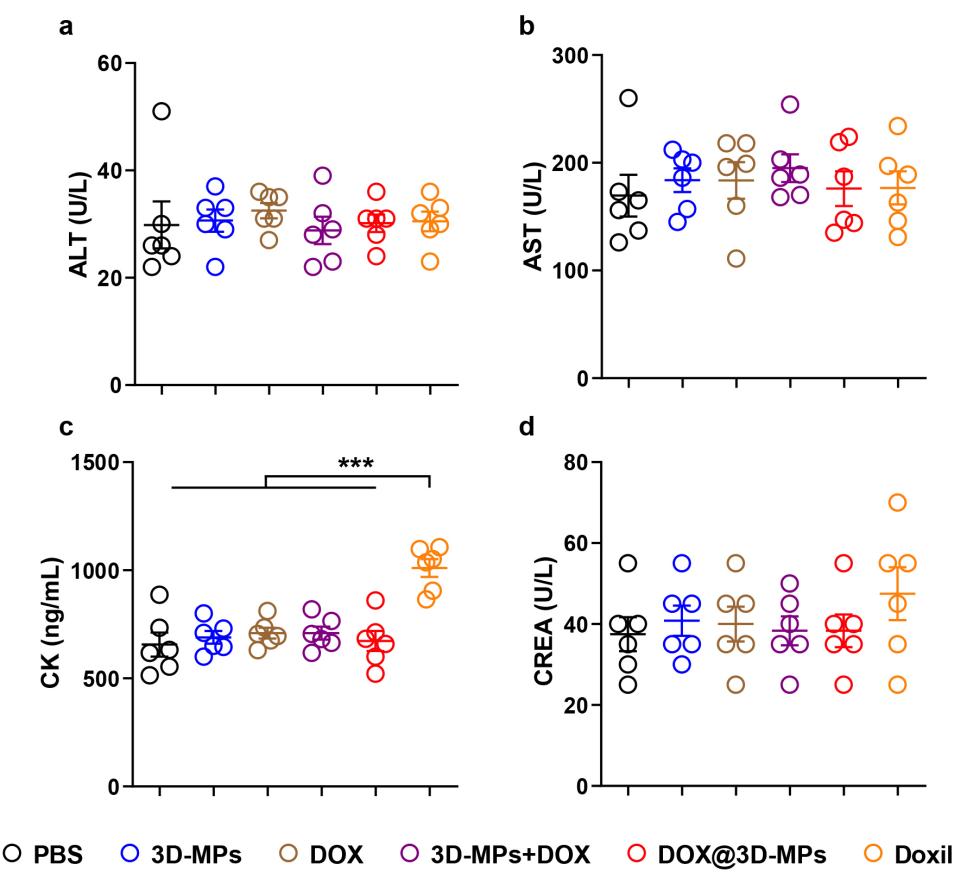


**Supplementary Fig. 6** **Serological analysis of subcutaneous H22 tumor-bearing mice after treatment with DOX@3D-MPs. a-d** Serological analysis of alanine aminotransferase (ALT, **a**), aspartate aminotransferase (AST, **b**), creatine kinase (CK, **c**) and creatinine (CREA, **d**) in H22 tumor-bearing mice at 11 days after intravenous injection of PBS, 3D-MPs, DOX, 3D-MPs+DOX, DOX@3D-MPs derived from H22 TRCs at DOX dosage of 0.75 mg kg^−1^ once every other day for 6 times, or high dosage of Doxil at 4 mg kg^−1^ once every three days for 3 times. Data are presented as means ± s.d. (n = 6 mice per group). ****P* < 0.001.


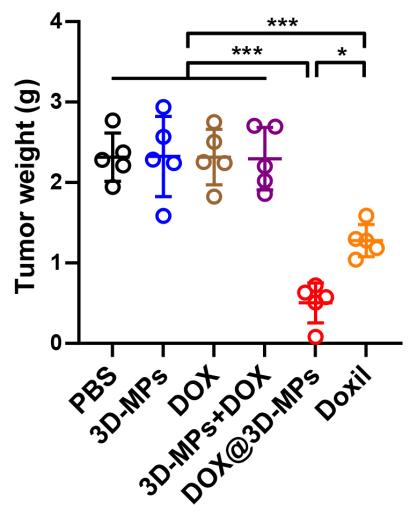


**Supplementary Fig. 7** Tumor weights of orthotopic 4T1 tumor-bearing mice at 11 days after intravenous injection of PBS, 3D-MPs, DOX, 3D-MPs+DOX, DOX@3D-MPs derived from 4T1 TRCs at DOX dosage of 0.75 mg kg^−1^ once every other day for 6 times, or high dosage of Doxil at 4 mg kg^−1^ once every three days for 3 times. Data are presented as means ± s.d. (n = 5 mice per group). **P* < 0.05, ****P* < 0.001.


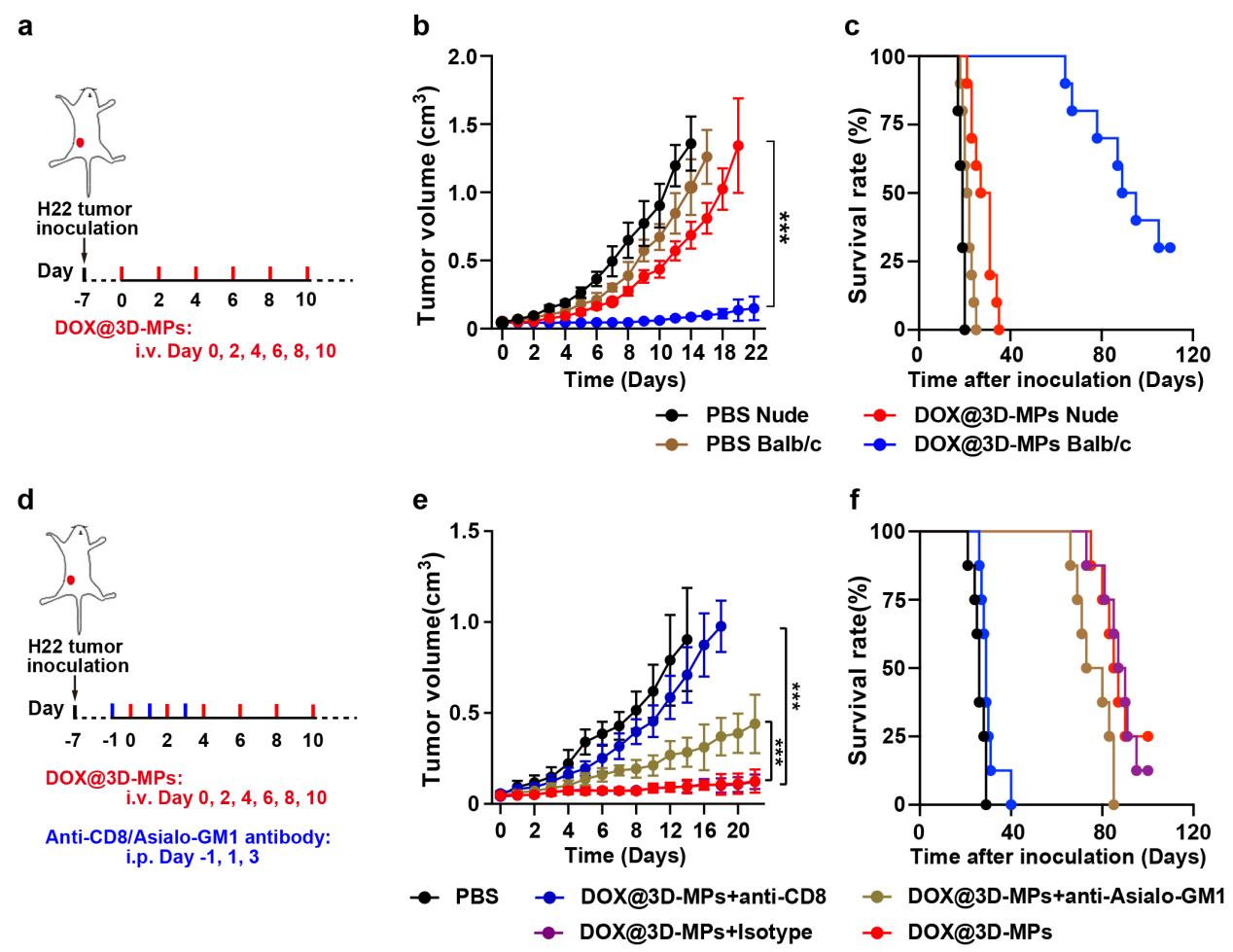


**Supplementary Fig. 8 Involvement of antitumor immunity in the antitumor effects of DOX@3D-MPs.** **a** Schematic schedule for the antitumor experiments in subcutaneous H22 tumor-bearing Balb/c mice and nude mice after intravenous injection of PBS or DOX@3D-MPs at DOX dosage of 0.75 mg kg^−1^ once every other day for 6 times. **b, c** Tumor growth curves (**b**) and Kaplan-Meier survival plots (**c**) of H22 tumor-bearing Balb/c mice and nude mice after treatments indicated in **a**. Data are presented as means ± s.d. (n = 10 mice per group). **d** Schematic schedule for the antitumor experiments in H22 tumor-bearing Balb/c mice after intravenous injection of PBS or DOX@3D-MPs at DOX dosage of 0.75 mg kg^−1^ once every other day for 6 times in the presence or absence of intraperitoneal injection of isotype (100 μg per mouse), anti-CD8 antibody (100 μg per mouse) or anti-Asialo-GM1 antibody (20 μL per mouse) once every other day for 3 times. **e, f** Tumor growth curves (**e**) and Kaplan-Meier survival plots (**f**) of H22 tumor-bearing mice after treatments indicated in **d.** Data are presented as means ± s.d. (n = 8 mice per group). ****P* < 0.001.


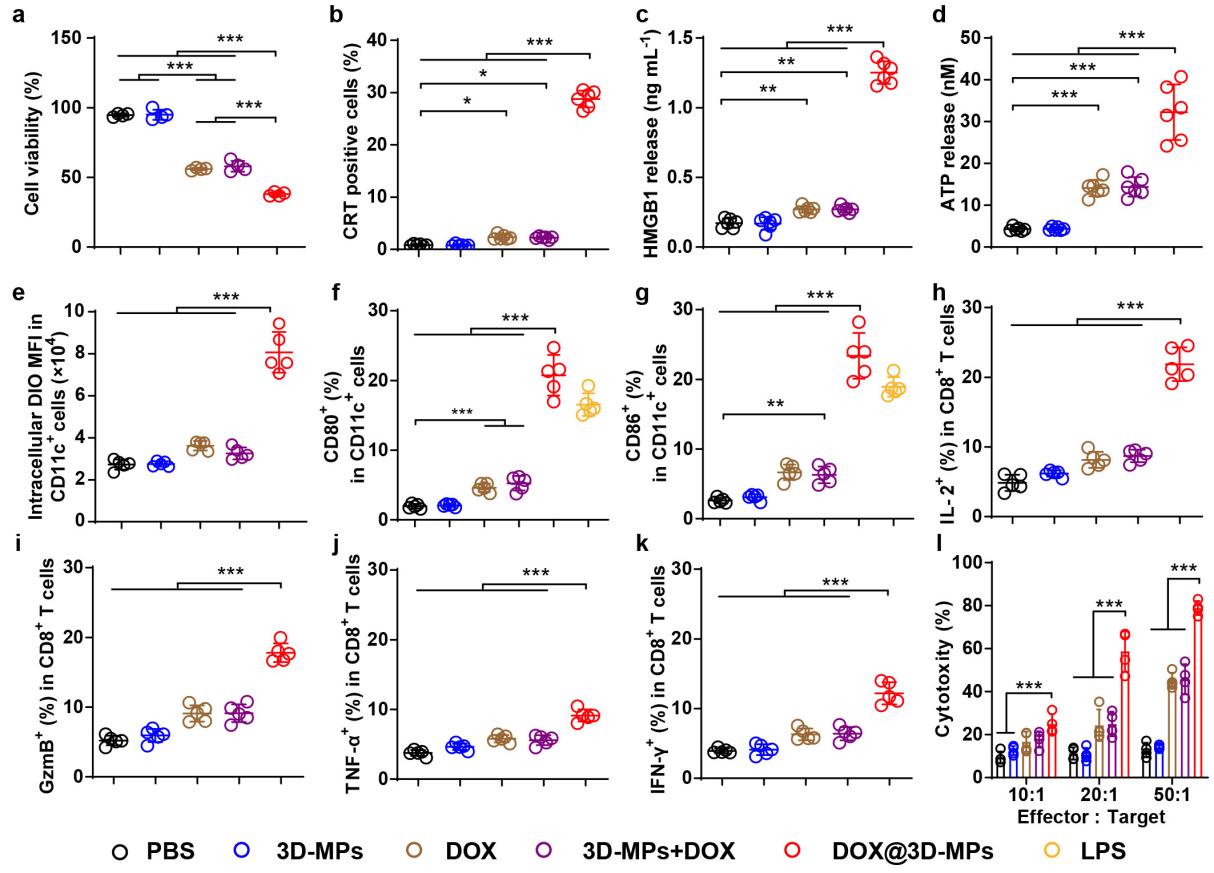


**Supplementary Fig. 9 In vitro antitumor immunity induced by DOX@3D-MPs**. **a** Cell viability of 4T1 cells at 24 h after treatment with PBS, 3D-MPs, DOX, 3D-MPs+DOX or DOX@3D-MPs derived from 4T1 TRCs at DOX concentration of 1 μg mL^−1^. Data are presented as means ± s.d. (n = 4 biologically independent samples). **b** CRT positive cell ratios of 4T1 cells at 12 h after treatments indicated in **a** by flow cytometry. Data are presented as means ± s.d. (n = 6 biologically independent samples). **c, d** HMGB1 (**c**) and ATP release (**d**) from 4T1 cells at 12 h after treatments indicated in **a**. Data are presented as means ± s.d. (n = 6 biologically independent samples). **e** Intracellular DiO MFI in CD11c^+^ BMDCs after immature BMDCs were co-cultured with the DiO-labeled 4T1 cells treated as indicated in **a** for 12 h by flow cytometry. Data are presented as means ± s.d. (n = 5 biologically independent samples). **f, g** Percentages of CD80^+^ (**f**) and CD86^+^ (**g**) cells in CD11c^+^ BMDCs at 12 h after immature BMDCs were co-cultured with 4T1 cells treated as indicated in **a** by flow cytometry. LPS (100 ng mL^−1^) was used as a positive control. Data are presented as means ± s.d. (n = 5 biologically independent samples). **h-k** Percentages of IL-2^+^ (**h**), GzmB^+^ (**i**), TNF-α^+^ (**j**) and IFN-γ^+^ (**k**) cells in CD8^+^ T cells after CD3^+^ T cells were incubated with the matured BMDCs treated as indicated in **f** for 3 days by flow cytometry. Data are presented as means ± s.d. (n = 5 biologically independent samples). **l** Cytotoxicity of activated T cells (effector cells) against 4T1 cells (target cells) at 24 h after the activated T cells treated as indicated in **h** were incubated with 4T1 cells at different effector/target ratios by LDH assay. Data are presented as means ± s.d. (n = 3 biologically independent samples).**P* < 0.05, ***P* < 0.01, ****P* < 0.001.


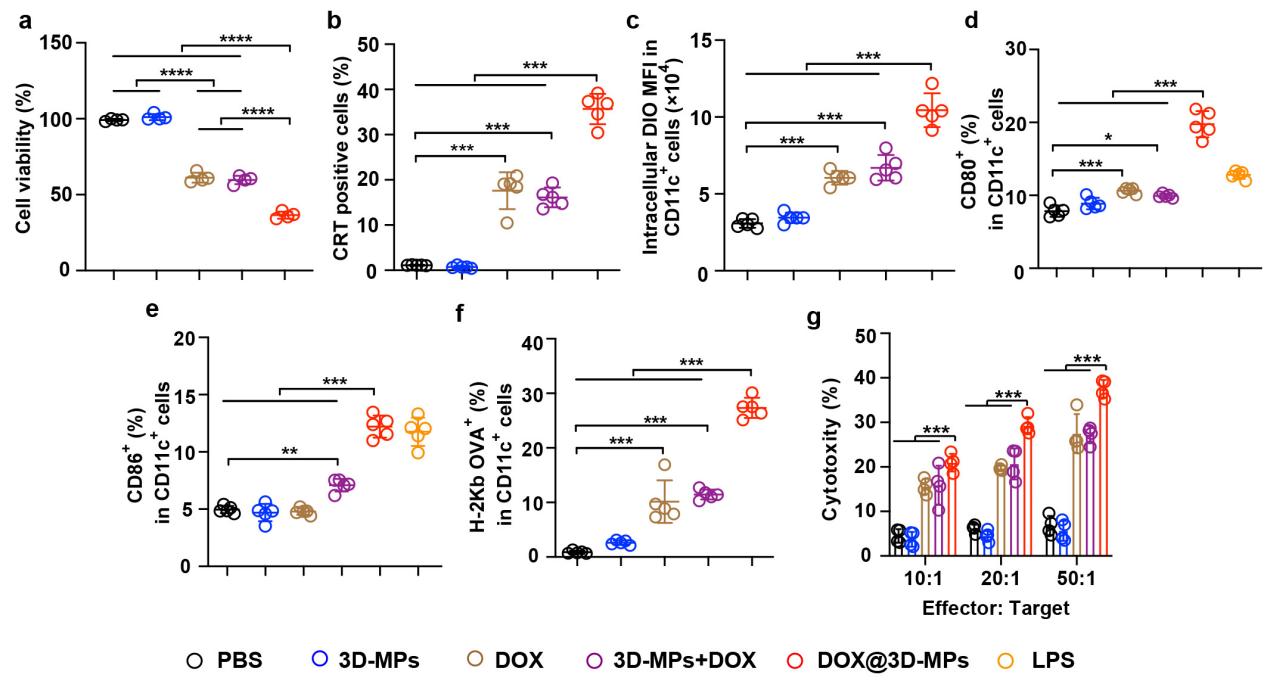


**Supplementary Fig. 10 In vitro antitumor immunity induced by DOX@3D-MPs.** **a** Cell viability of B16-OVA cells at 24 h after treatment with PBS, 3D-MPs, DOX, 3D-MPs+DOX or DOX@3D-MPs derived from B16 TRCs at DOX concentration of 1 μg mL^−1^. Data are presented as means ± s.d. (n = 4 biologically independent samples). **b** CRT positive cell ratios of B16-OVA cells at 12 h after treatments indicated in **a** by flow cytometry. Data are presented as means ± s.d. (n = 5 biologically independent samples). **c** Intracellular DiO MFI in CD11c^+^ BMDCs after immature BMDCs were co-cultured with the DiO-labeled B16-OVA cells treated as indicated in **a** for 12 h by flow cytometry. Data are presented as means ± s.d. (n = 5 biologically independent samples). **d-f** Percentages of CD80^+^ (**d**), CD86^+^ (**e**), H-2Kb OVA^+^ (**f**) cells in CD11c^+^ BMDCs at 12 h after immature BMDCs were co-cultured with B16-OVA cells treated as indicated in **a** by flow cytometry. LPS (100 ng mL^−1^) was used as a positive control. Data are presented as means ± s.d. (n = 5 biologically independent samples). **g** Cytotoxicity of activated T cells (effector cells) against B16-OVA cells (target cells) at 24 h after CD3^+^ T cells incubated with the matured BMDCs treated as indicated in **d** for 3 days were incubated with B16-OVA cells at different effector/target ratios by LDH assay. Data are presented as means ± s.d. (n = 3 biologically independent samples). **P* < 0.05, ***P* < 0.01, ****P* < 0.001.


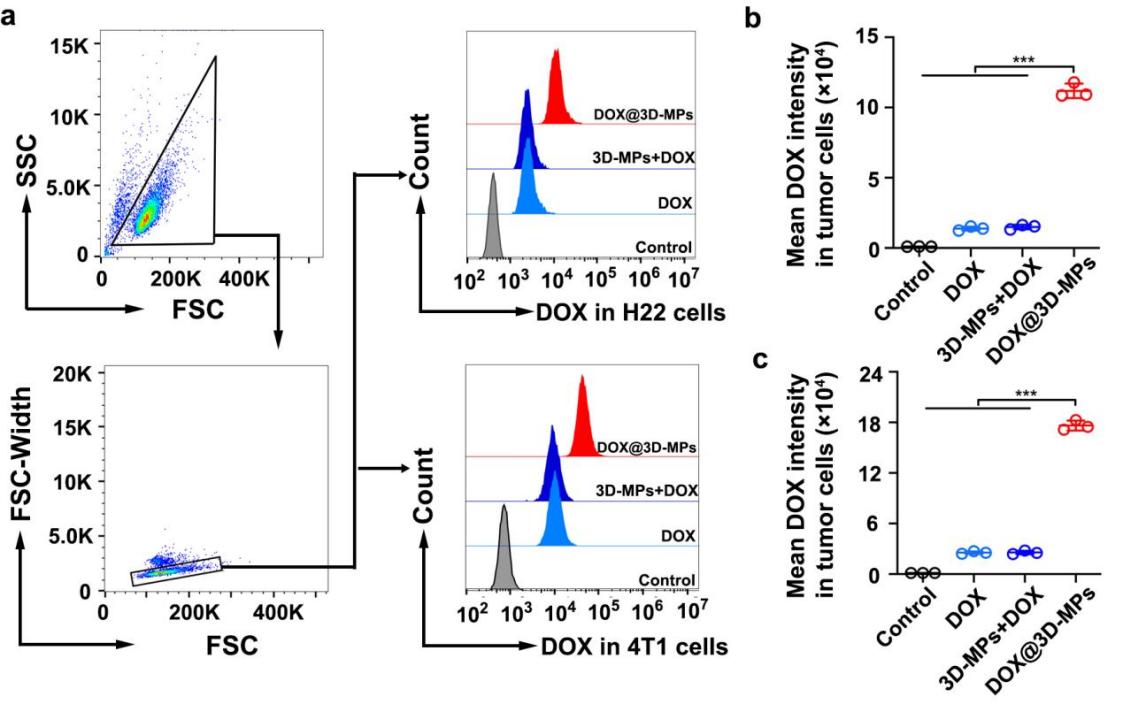


**Supplementary Fig. 11 Efficient cellular uptake of DOX@3D-MPs by tumor cells. a** Gating strategy for analyzing the mean DOX fluorescence intensity in H22 and 4T1 cells after treatment with DOX, 3D-MPs+DOX or DOX@3D-MPs derived from H22 TRCs and 4T1 TRCs at DOX concentration of 1 μg mL^−1^ for 4 h by flow cytometry, respectively. **b, c** Quantification of mean DOX fluorescence intensity in H22 (**b**) and 4T1 (**c**) cells after treatment indicated in **a**. Data are presented as means ± s.d. (n = 3 biologically independent samples). ****P* < 0.001.


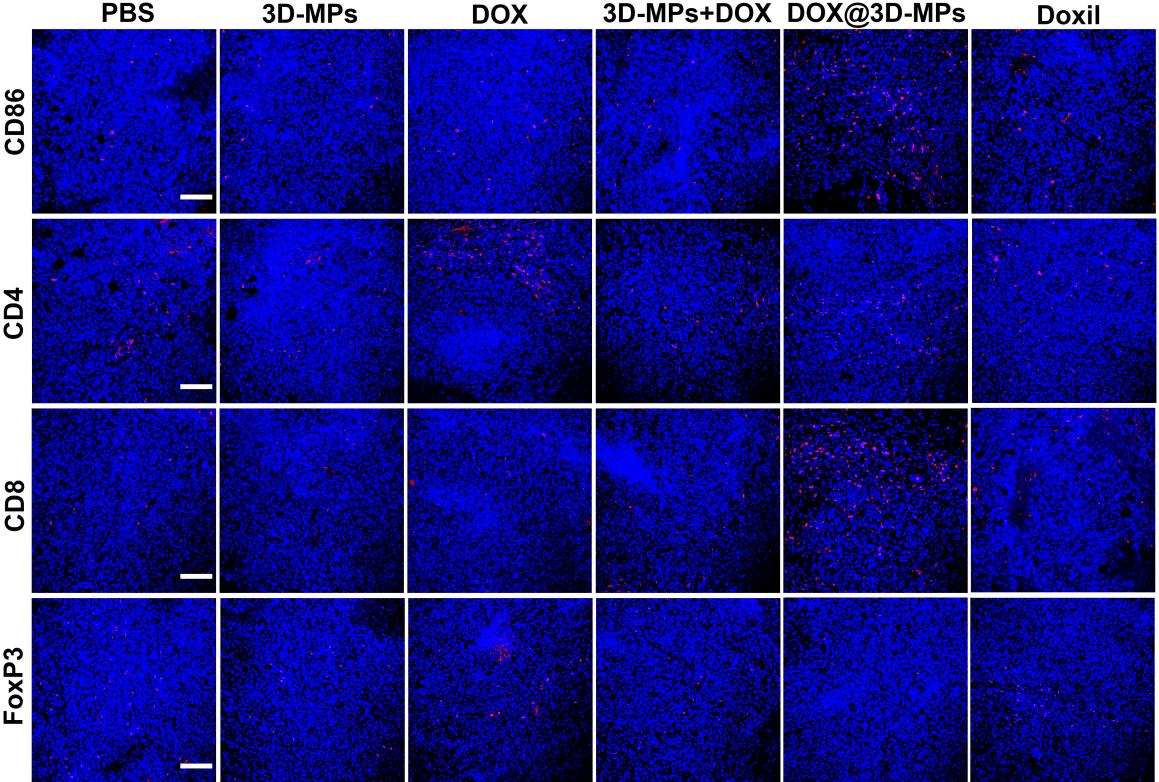


**Supplementary Fig. 12 DOX@3D-MPs-induced improved tumor immune microenvironment in subcutaneous H22 tumor-bearing mice.** Representative immunofluorescence images of CD86-, CD4-, CD8-, FoxP3-stained (red) tumor tissues of H22 tumor-bearing mice at 11 days after intravenous injection of PBS, 3D-MPs, DOX, 3D-MPs+DOX, DOX@3D-MPs at DOX dosage of 0.75 mg kg^−1^ once every other day for 6 times, or high dosage of Doxil at 4 mg kg^−1^ once every three days for 3 times by confocal microscope. Scale bar: 200 μm.


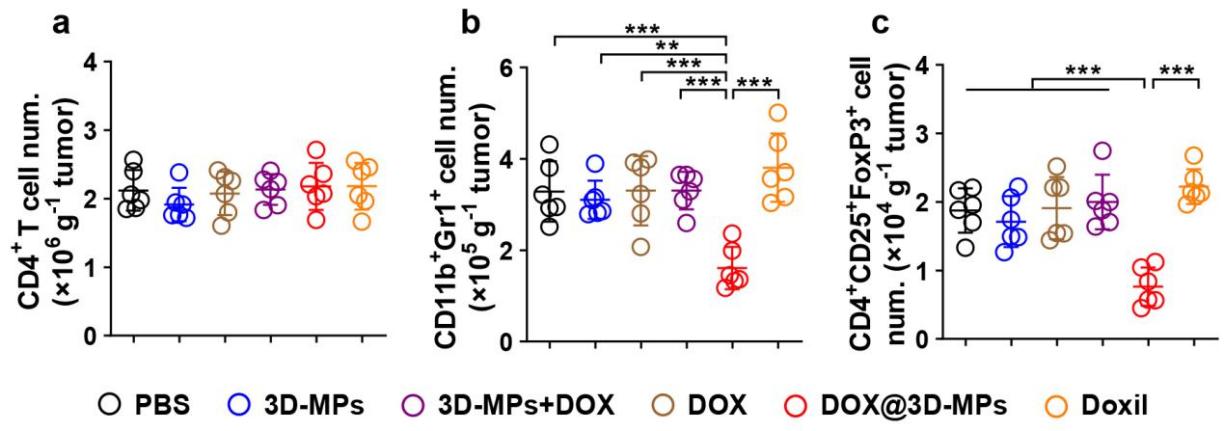


**Supplementary Fig. 13 DOX@3D-MPs-induced tumor immune microenvironment in subcutaneous H22 tumor-bearing mice. a-c** Numbers of CD4^+^ T (**a**), CD11b^+^Gr1^+^ (**b**) and CD4^+^CD25^+^FoxP3^+^ cells (**c**) in tumor tissues of H22 tumor-bearing mice at 11 days after intravenous injection of PBS, 3D-MPs, DOX, 3D-MPs+DOX, DOX@3D-MPs derived from H22 TRCs at DOX dosage of 0.75 mg kg^−1^ once every other day for 6 times, or high dosage of Doxil at 4 mg kg^−1^ once every three days for 3 times. Data are presented as means ± s.d. (n = 6 mice per group). ****P* < 0.001.


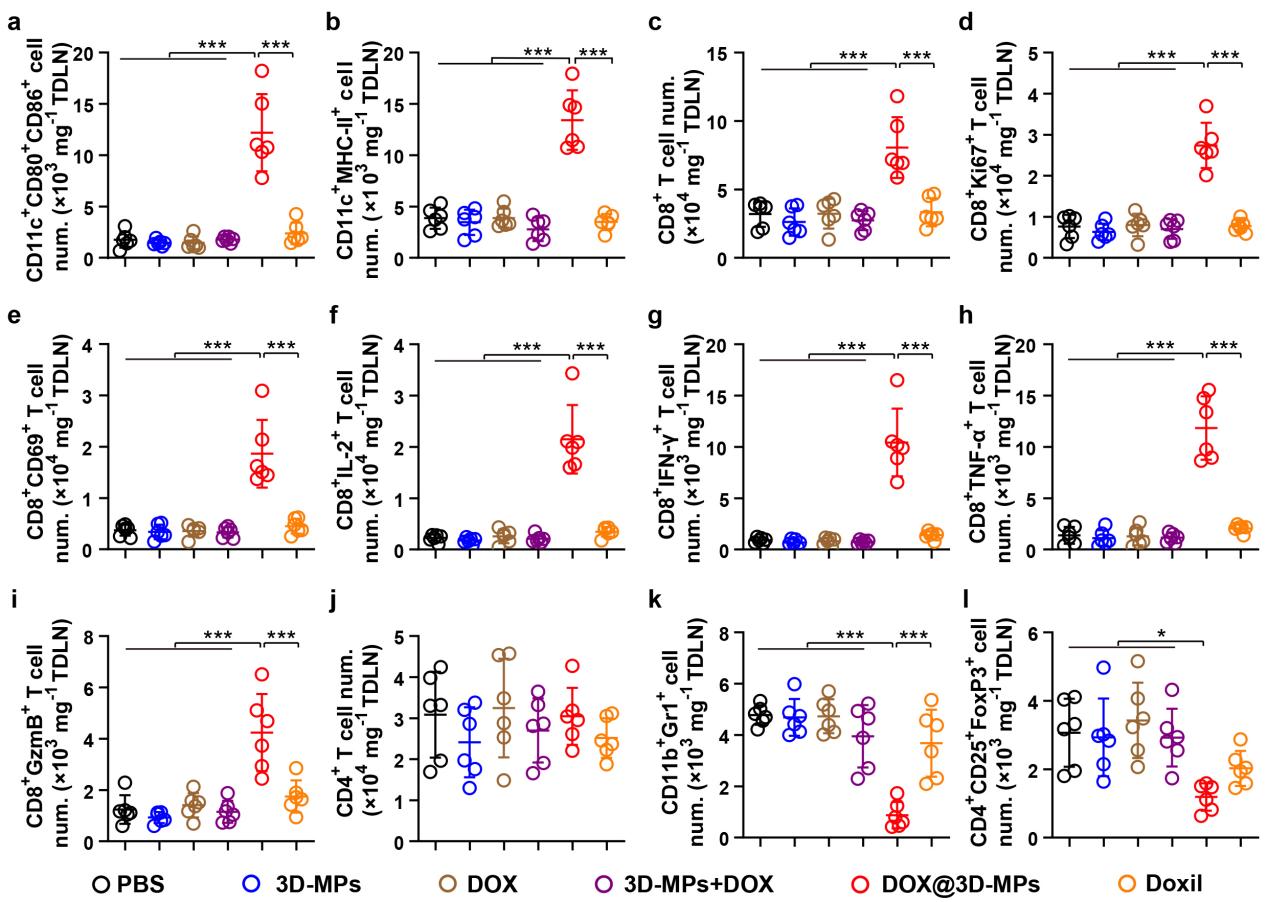


**Supplementary Fig. 14 DOX@3D-MPs-induced improved immune microenvironment in** **tumor draining lymph nodes of subcutaneous H22 tumor-bearing mice. a-l** Numbers of CD11c^+^CD80^+^CD86^+^ (**a**), CD11c^+^MHC-II^+^ (**b**), CD8^+^ T (**c**), CD8^+^Ki67^+^ T (**d**), CD8^+^CD69^+^ T (**e**), CD8^+^IL-2^+^ T (**f**), CD8^+^IFN-γ^+^ T (**g**), CD8^+^TNF-α^+^ T (**h**), CD8^+^GzmB^+^ T (**i**), CD4^+^ T (**j**), CD11b^+^Gr1^+^ (**k**) and CD4^+^CD25^+^FoxP3^+^ (**l**) cells in tumor draining lymph nodes of H22 tumor-bearing mice at 11 days after intravenous injection of PBS, 3D-MPs, DOX, 3D-MPs+DOX, DOX@3D-MPs derived from H22 TRCs at DOX dosage of 0.75 mg kg^−1^ once every other day for 6 times, or high dosage of Doxil at 4 mg kg^−1^ once every three days for 3 times by flow cytometry. Data are presented as means ± s.d. (n = 6 mice per group). **P* < 0.05, ****P* < 0.001.


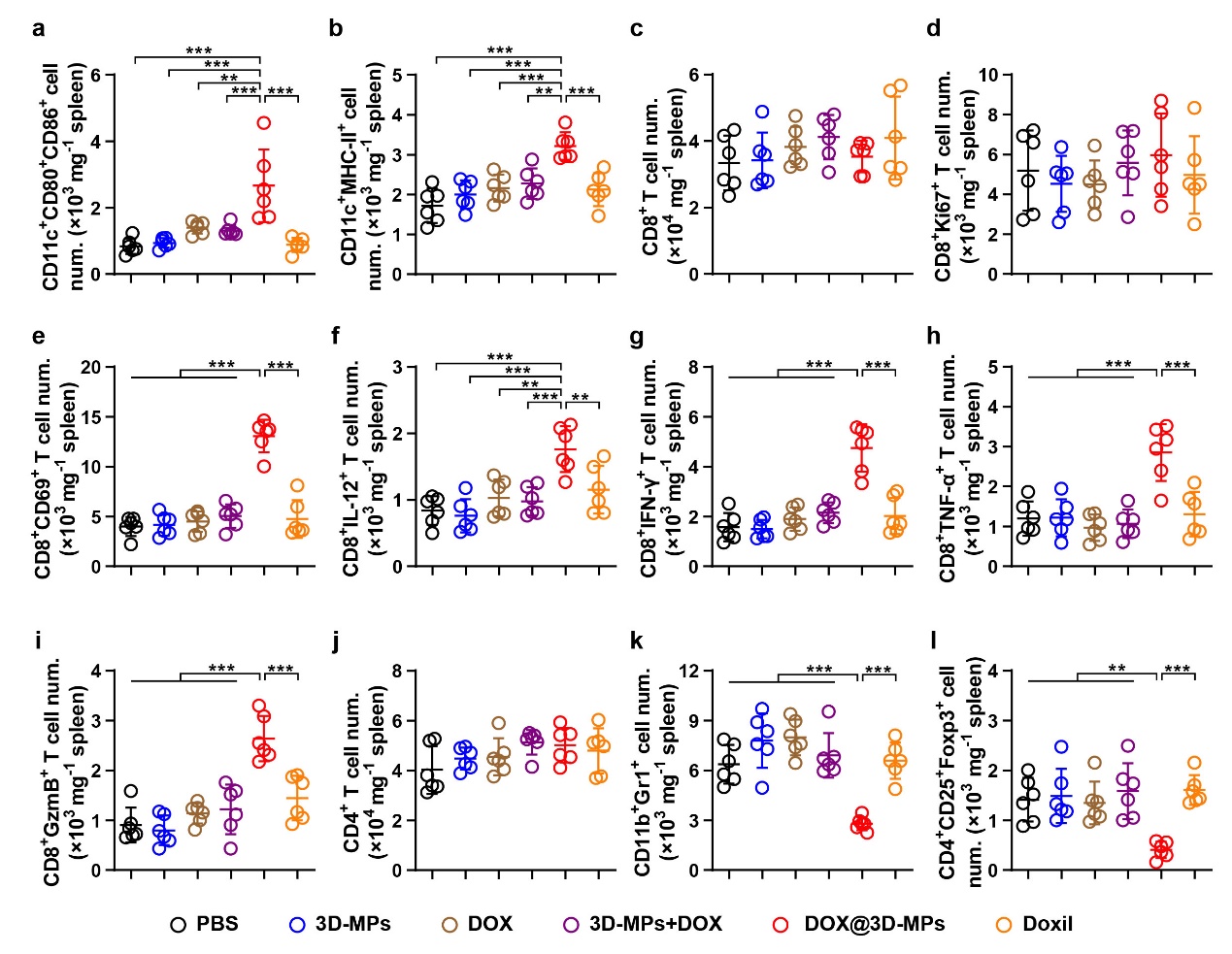


**Supplementary Fig. 15 DOX@3D-MPs-induced improved immune microenvironment in spleens of subcutaneous H22 tumor-bearing mice. a-l** Numbers of CD11c^+^CD80^+^CD86^+^ (**a**), CD11c^+^MHC-II^+^ (**b**), CD8^+^ T (**c**), CD8^+^Ki67^+^ T (**d**), CD8^+^CD69^+^ T (**e**), CD8^+^IL-2^+^ T (**f**), CD8^+^IFN-γ^+^ T (**g**), CD8^+^TNF-α^+^ T (**h**), CD8^+^GzmB^+^ T (**i**), CD4^+^ T (**j**), CD11b^+^Gr1^+^ (**k**) and CD4^+^CD25^+^FoxP3^+^ (**l**) cells in spleens of H22 tumor-bearing mice at 11 days after intravenous injection of PBS, 3D-MPs, DOX, 3D-MPs+DOX, DOX@3D-MPs derived from H22 TRCs at DOX dosage of 0.75 mg kg^−1^ once every other day for 6 times, or high dosage of Doxil at 4 mg kg^−1^ once every three days for 3 times by flow cytometry. Data are presented as means ± s.d. (n = 6 mice per group). ***P* < 0.01, ****P* < 0.001.

**
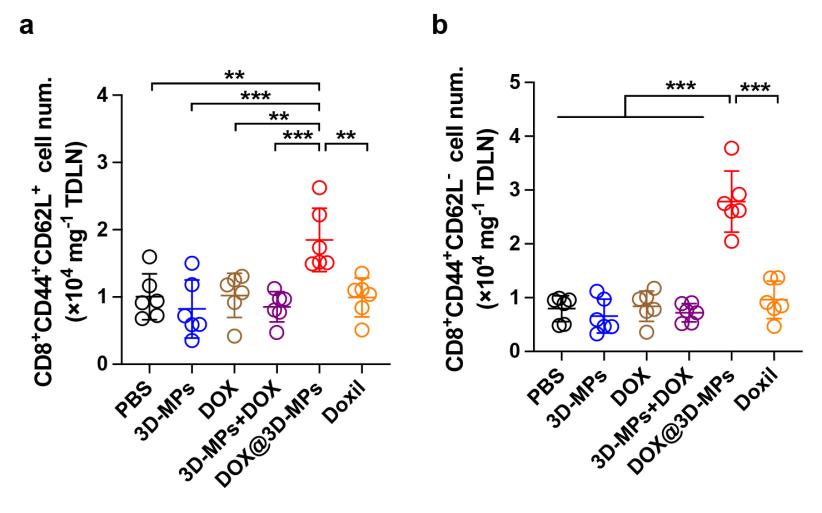
**

**Supplementary Fig. 16 DOX@3D-MPs-induced enhanced immune memory in subcutaneous H22 tumor-bearing mice. a, b** Numbers of CD8^+^CD44^+^CD62L^+^ T (**a**) and CD8^+^CD44^+^CD62L^-^ T (**b**) cells in tumor draining lymph nodes of subcutaneous H22 tumor-bearing mice at 11 days after intravenous injection of PBS, 3D-MPs, DOX, 3D-MPs+DOX, DOX@3D-MPs at DOX dosage of 0.75 mg kg^−1^ once every other day for 6 times, or high dosage of Doxil at 4 mg kg^−1^ once every three days for 3 times by flow cytometry. Data are presented as means ± s.d. (n = 6 mice per group). ***P* < 0.01, ****P* < 0.001.


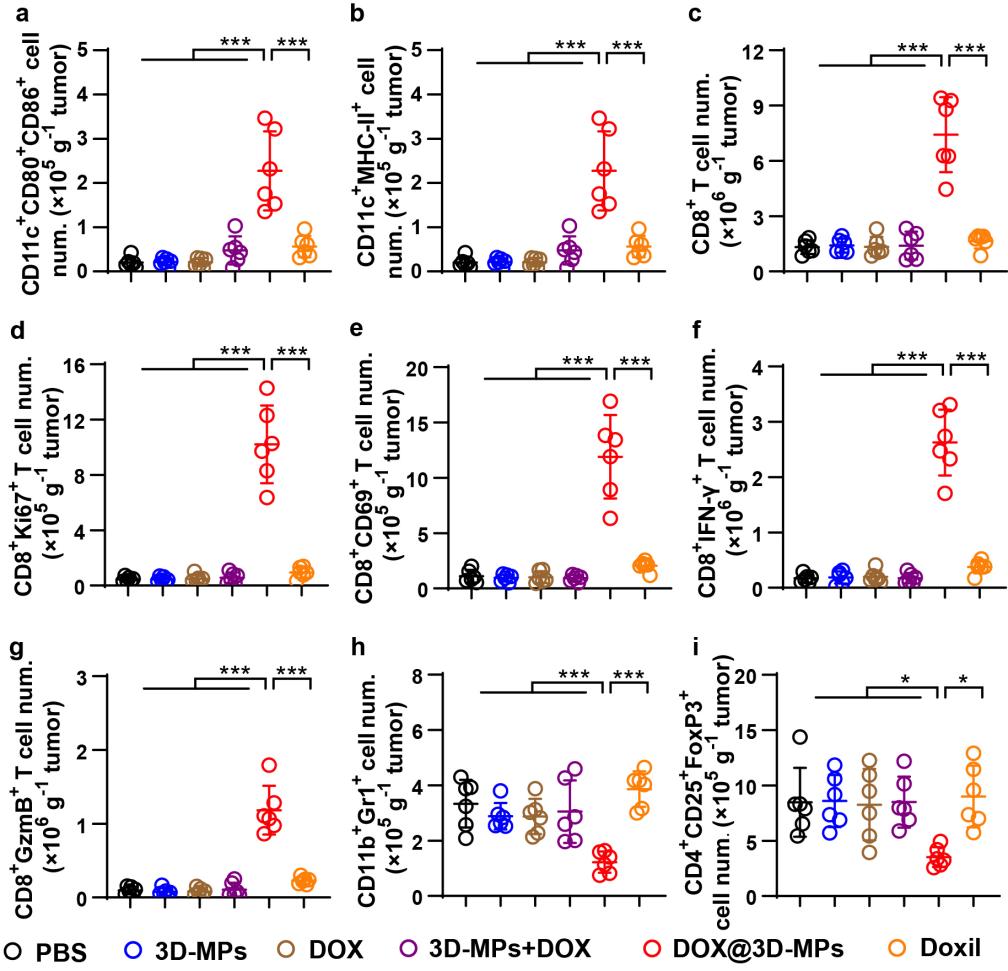


**Supplementary Fig. 17 DOX@3D-MPs-induced improved tumor immune microenvironment in orthotopic 4T1 tumor-bearing mice. a-j** Numbers of CD11c^+^CD80^+^CD86^+^ (**a**), CD11c^+^MHC-II^+^ (**b**), CD8^+^ T (**c**), CD8^+^Ki67^+^ T (**d**), CD8^+^CD69^+^ T (**e**), CD8^+^IFN-γ^+^ T (**f**), CD8^+^GzmB^+^ T (**g**), CD11b^+^Gr1^+^ (**h**) and CD4^+^CD25^+^FoxP3^+^ (**i**) cells in tumor tissues of 4T1 tumor-bearing mice at 11 days after intravenous injection of PBS, 3D-MPs, DOX, 3D-MPs+DOX, DOX@3D-MPs derived from 4T1 TRCs at DOX dosage of 0.75 mg kg^−1^ once every other day for 6 times, or high dosage of Doxil at 4 mg kg^−1^ once every three days for 3 times indicated in Fig. **4m** by flow cytometry. Data are presented as means ± s.d. (n = 6 mice per group). **P* < 0.05, ****P* < 0.001.


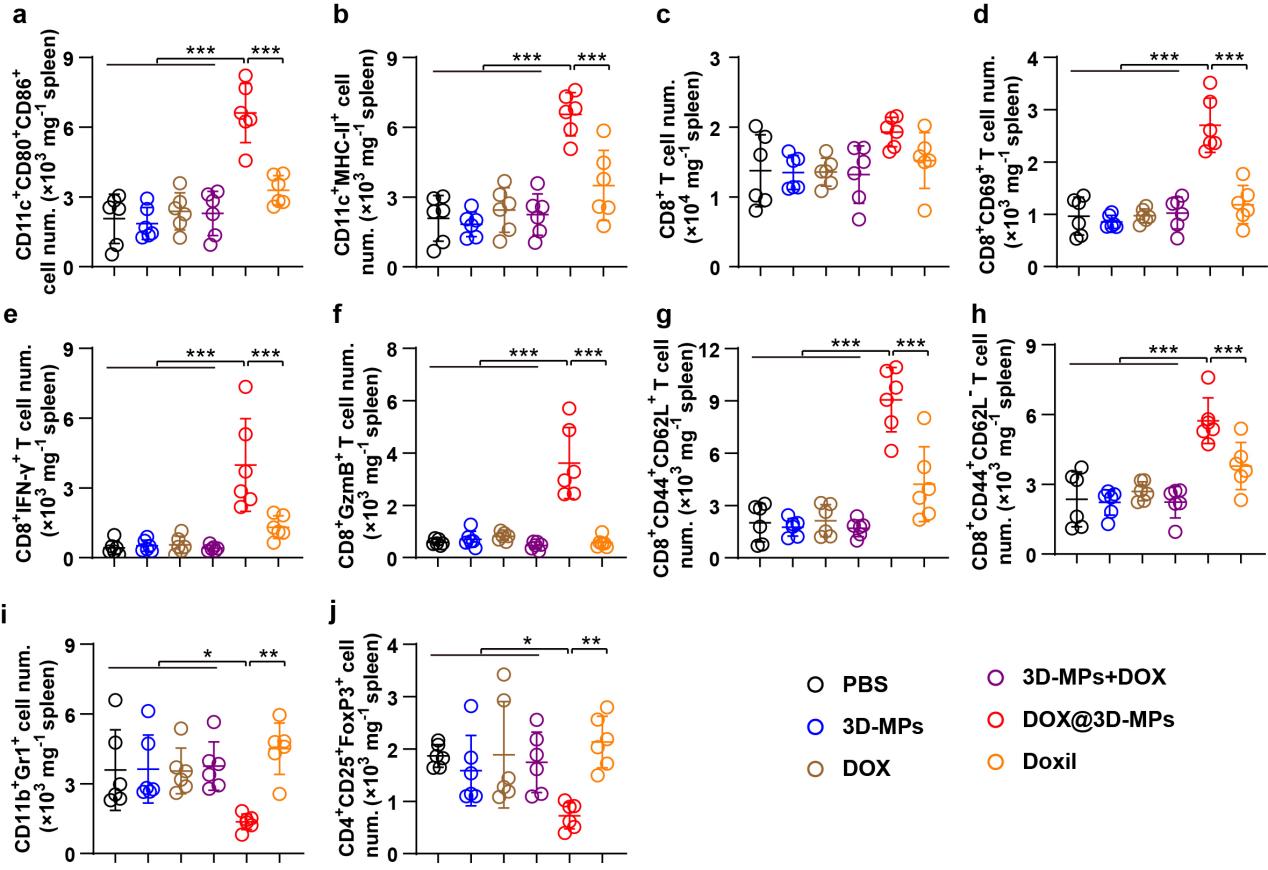


**Supplementary Fig. 18 DOX@3D-MPs-induced improved immune microenvironment in spleens of orthotopic 4T1 tumor-bearing mice. a-k** Numbers of CD11c^+^CD80^+^CD86^+^ (**a**), CD11c^+^MHC-II^+^ (**b**), CD8^+^ T (**c**), CD8^+^CD69^+^ T (**d**), CD8^+^IFN-γ^+^ T (**e**), CD8^+^GzmB^+^ T (**f**), CD8^+^CD44^+^CD62L^+^ T (**g**), CD8^+^CD44^+^CD62L^-^ T (**h**), CD11b^+^Gr1^+^ (**i**) and CD4^+^CD25^+^FoxP3^+^ (**j**) cells in spleens of 4T1 tumor-bearing mice at 11 days after intravenous injection of PBS, 3D-MPs, DOX, 3D-MPs+DOX, DOX@3D-MPs derived from 4T1 TRCs at DOX dosage of 0.75 mg kg^−1^ once every other day for 6 times, or high dosage of Doxil at 4 mg kg^−1^ once every three days for 3 times indicated in Fig. **4m** by flow cytometry. Data are presented as means ± s.d. (n = 6 mice per group). **P* < 0.05, ***P* < 0.01, ****P* < 0.001.

**
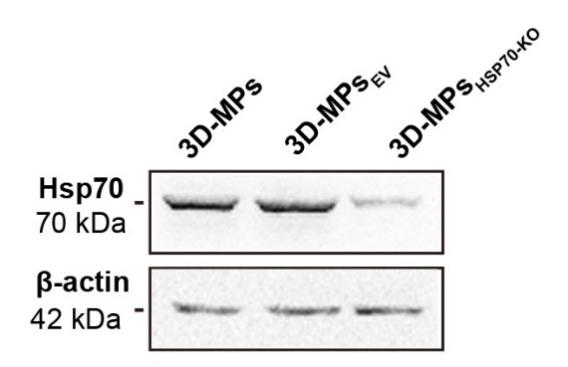
**

**Supplementary Fig. 19** HSP70 expression in 3D-MPs, 3D-MPs_EV_ and 3D-MPs_HSP70-KO_ derived from H22 TRCs, H22 TRCs stably expressing empty vector and H22 TRCs stably knocking out HSP70 by western blotting analysis, respectively.

**
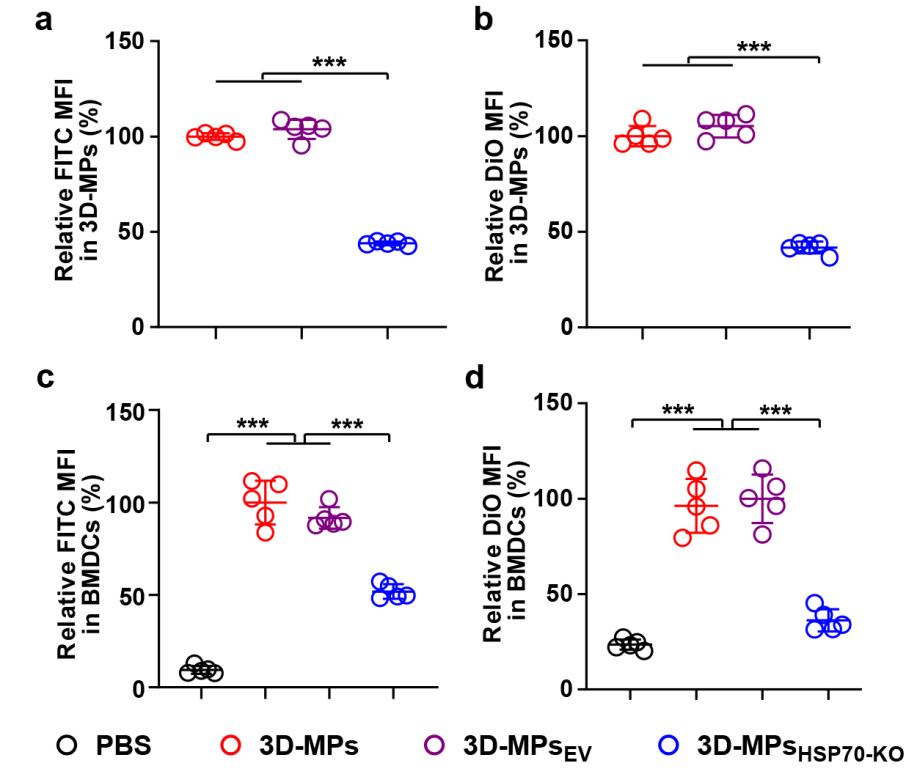
**

**Supplementary Fig. 20 Effects of HSP70 in 3D-MPs on antigen phagocytosis by BMDCs. a, b** Relative FITC (**a**) and DiO (**b**) MFI after 3D-MPs, 3D-MPs_EV_ or 3D-MPs_HSP70-KO_ (10 μg mL^−1^) were incubated with FITC-labeled OVA or DiO-labeled H22 cell debris (1 μg mL^−1^) for 1 h by flow cytometry, respectively. Data are presented as means ± s.d. (n = 5 biologically independent samples). **c, d** Relative FITC (**c**) and DiO (**d**) MFI in BMDCs at 12 h after BMDCs were incubated with PBS, 3D-MPs, 3D-MPs_EV_ or 3D-MPs_HSP70-KO_ in the presence of FITC-labeled OVA protein or DiO-labeled H22 cell debris by flow cytometry, respectively. Data are presented as means ± s.d. (n = 5 biologically independent samples). ****P* < 0.001.


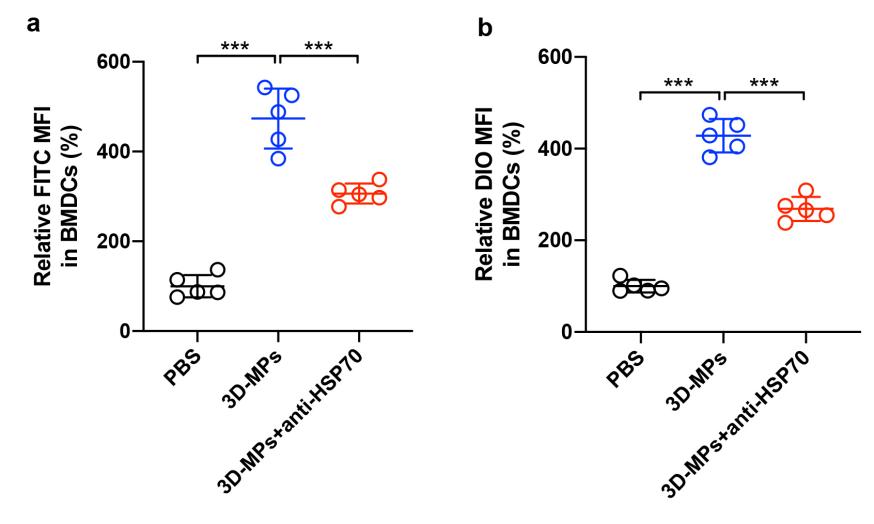


**Supplementary Fig. 21 Effects of HSP70 in 3D-MPs on antigen phagocytosis by BMDCs. a, b** Relative FITC (**a**) and DiO (**b**) MFI in BMDCs at 12 h after BMDCs were incubated with PBS, 3D-MPs or 3D-MPs pretreated with anti-HSP70 antibody (denoted as 3D-MPs+anti-HSP70) in the presence of FITC-labeled OVA protein or DiO-labeled H22 cell debris by flow cytometry, respectively. Data are presented as means ± s.d. (n = 5 biologically independent samples). ****P* < 0.001.

**
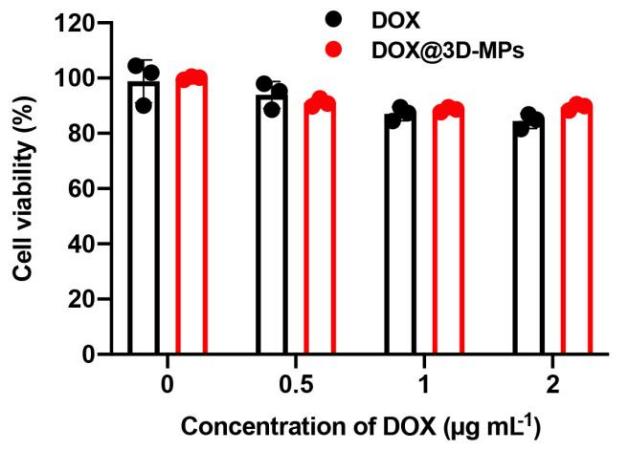
**

**Supplementary Fig. 22** Cell viability of BMDCs after treatment with DOX or DOX@3D-MP derived from H22 TRCs at different concentrations of DOX for 24 h. Data are presented as means ± s.d. (n = 3 biologically independent samples).


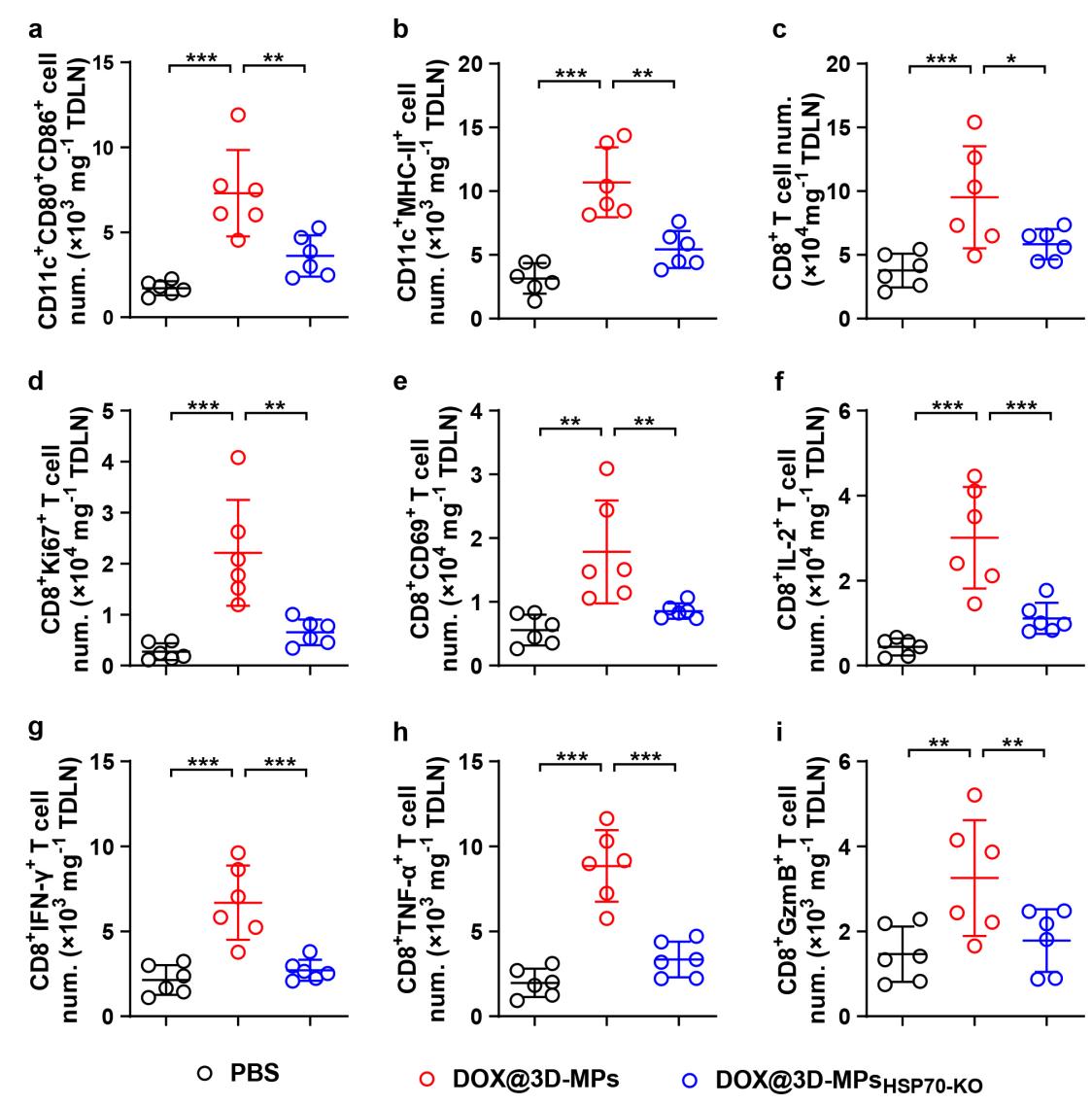


**Supplementary Fig. 23** **Effects of HSP70 in 3D-MPs on DOX@3D-MPs-improved immune microenvironment in tumor draining lymph nodes of subcutaneous H22 tumor-bearing mice. a-i** Numbers of CD11c^+^CD80^+^CD86^+^ (**a**), CD11c^+^MHC-II^+^ (**b**), CD8^+^ T (**c**), CD8^+^Ki67^+^ T (**d**), CD8^+^CD69^+^ T (**e**), CD8^+^IL-2^+^ T (**f**), CD8^+^IFN-γ^+^ T (**g**), CD8^+^TNF-α^+^ T (**h**) and CD8^+^GzmB^+^ T (**i**) cells in tumor draining lymph nodes of H22 tumor-bearing mice at 11 days after intravenous injection of PBS, DOX@3D-MPs or DOX@3D-MPs_HSP70-KO_ at DOX dosage of 0.75 mg kg^−1^ once every other day for 6 times by flow cytometry. Data are presented as means ± s.d. (n = 6 mice per group). **P* < 0.05, ***P* < 0.01, ****P* < 0.001.


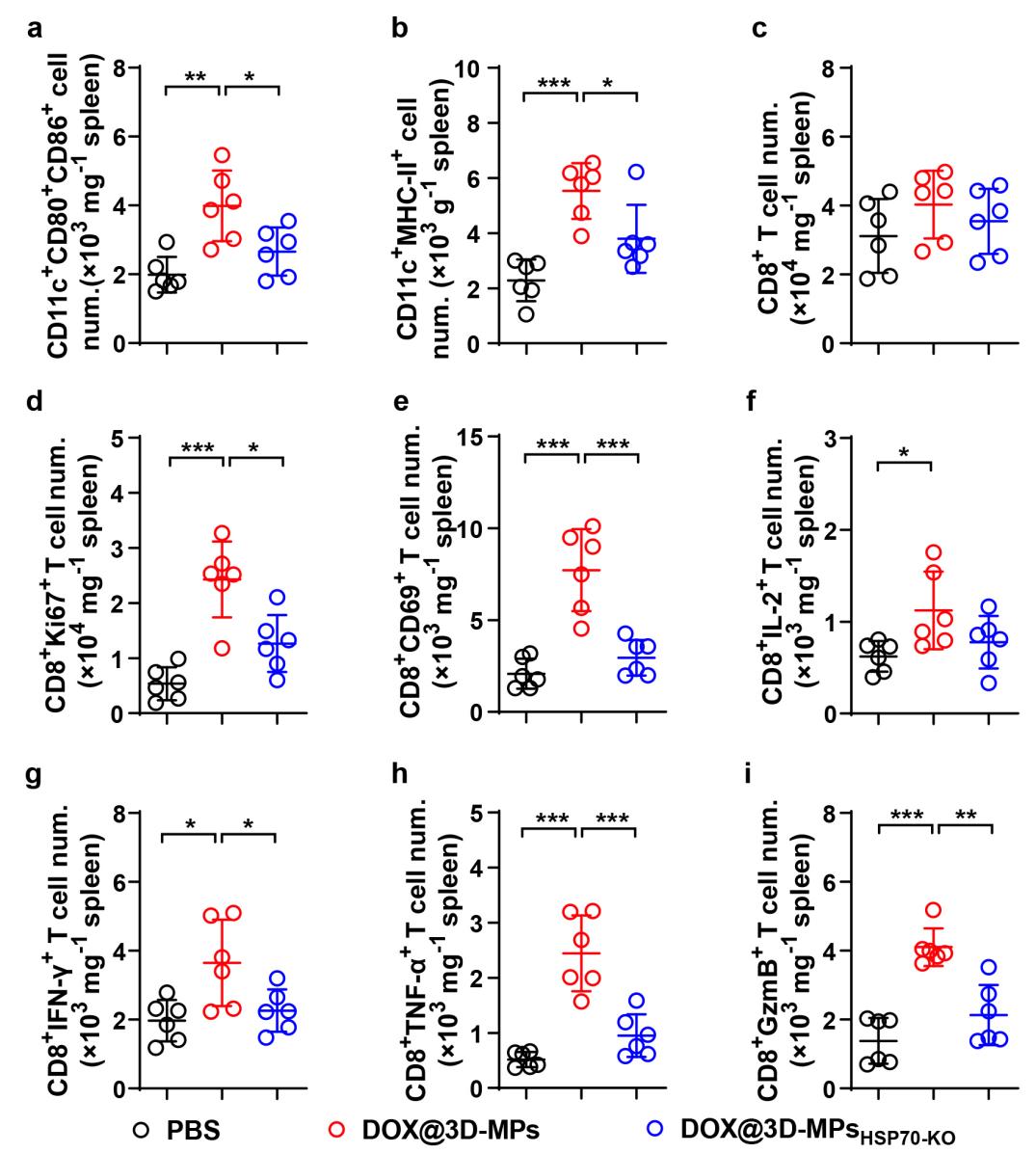


**Supplementary Fig. 24 Effects of HSP70 in 3D-MPs on DOX@3D-MPs-improved immune microenvironment in spleens of subcutaneous H22 tumor-bearing mice. a-i** Numbers of CD11c^+^CD80^+^CD86^+^ (**a**), CD11c^+^MHC-II^+^ (**b**), CD8^+^ T (**c**), CD8^+^Ki67^+^ T (**d**), CD8^+^CD69^+^ T (**e**), CD8^+^IL-2^+^ T (**f**), CD8^+^IFN-γ^+^ T (**g**), CD8^+^TNF-α^+^ T (**h**) and CD8^+^GzmB^+^ T (**i**) cells in spleens of H22 tumor-bearing mice at 11 days after intravenous injection of PBS, DOX@3D-MPs or DOX@3D-MPs_HSP70-KO_ at DOX dosage of 0.75 mg kg^−1^ once every other day for 6 times by flow cytometry. Data are presented as means ± s.d. (n = 6 mice per group). **P* < 0.05, ***P* < 0.01, ****P* < 0.001.


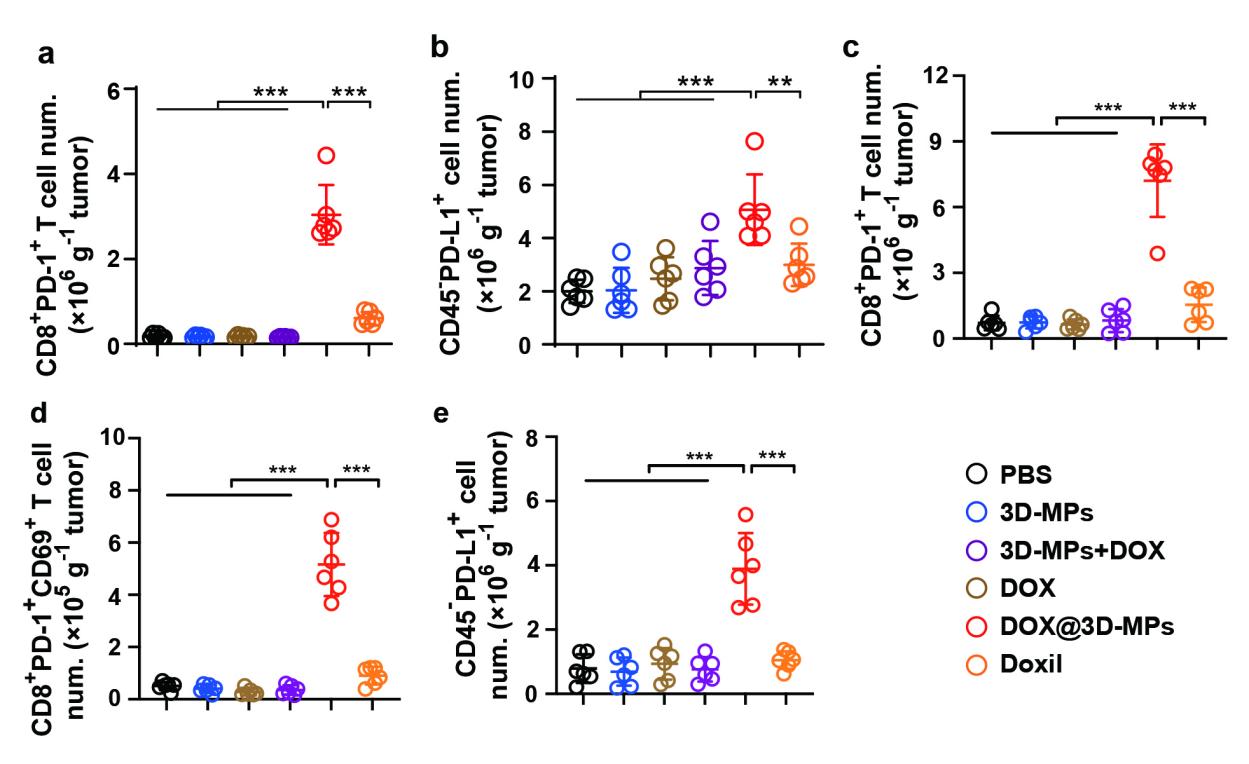


**Supplementary Fig. 25 PD-1 and PD-L1 expression in tumor tissues of subcutaneous H22 and orthotopic 4T1 tumor-bearing mice after treatment with DOX@3D-MPs. a, b** Numbers of CD8^+^PD-1^+^ T (**a**) and CD45^-^PD-L1^+^ (**b**) cells in tumor tissues of H22 tumor-bearing mice at 11 days after intravenous injection of PBS, 3D-MPs, DOX, 3D-MPs+DOX, DOX@3D-MPs at DOX dosage of 0.75 mg kg^−1^ once every other day for 6 times, or high dosage of Doxil at 4 mg kg^−1^ once every three days for 3 times by flow cytometry. **c-e** Numbers of CD8^+^PD-1^+^ T (**c**), CD8^+^PD-1^+^CD69^+^ T (**d**) and CD45^-^PD-L1^+^ (**e**) cells in tumor tissues of 4T1 tumor-bearing mice at 11 days after treatment indicated in **a** by flow cytometry. Data are presented as means ± s.d. (n = 6 mice per group). ***P* < 0.01, ****P* < 0.001.


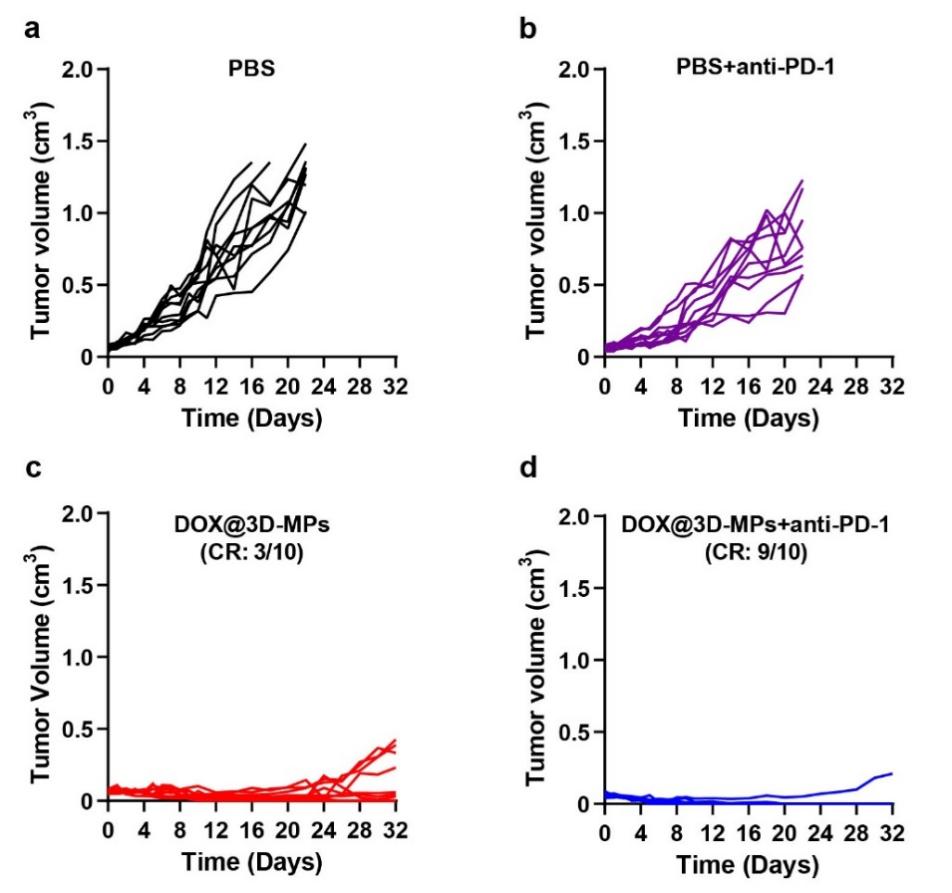


**Supplementary Fig. 26 Potent anticancer activity of combination of DOX@3D-MPs and anti-PD-1 antibody in subcutaneous H22 tumor-bearing mice. a-d** Individual tumor growth curves of H22 tumor-bearing mice after treatment with PBS (**a**), a combination of PBS and anti-PD-1 antibody (**b**), DOX@3D-MPs derived from H22 TRCs (**c**) or a combination of DOX@3D-MPs and anti-PD-1 antibody (**d**) at DOX dosages of 0.75 mg kg^−1^ once every other day for six times and intraperitoneal injection of anti-PD-1 antibody at dosage of 5 mg kg^−1^ every four days for three times (n = 10 mice per group).


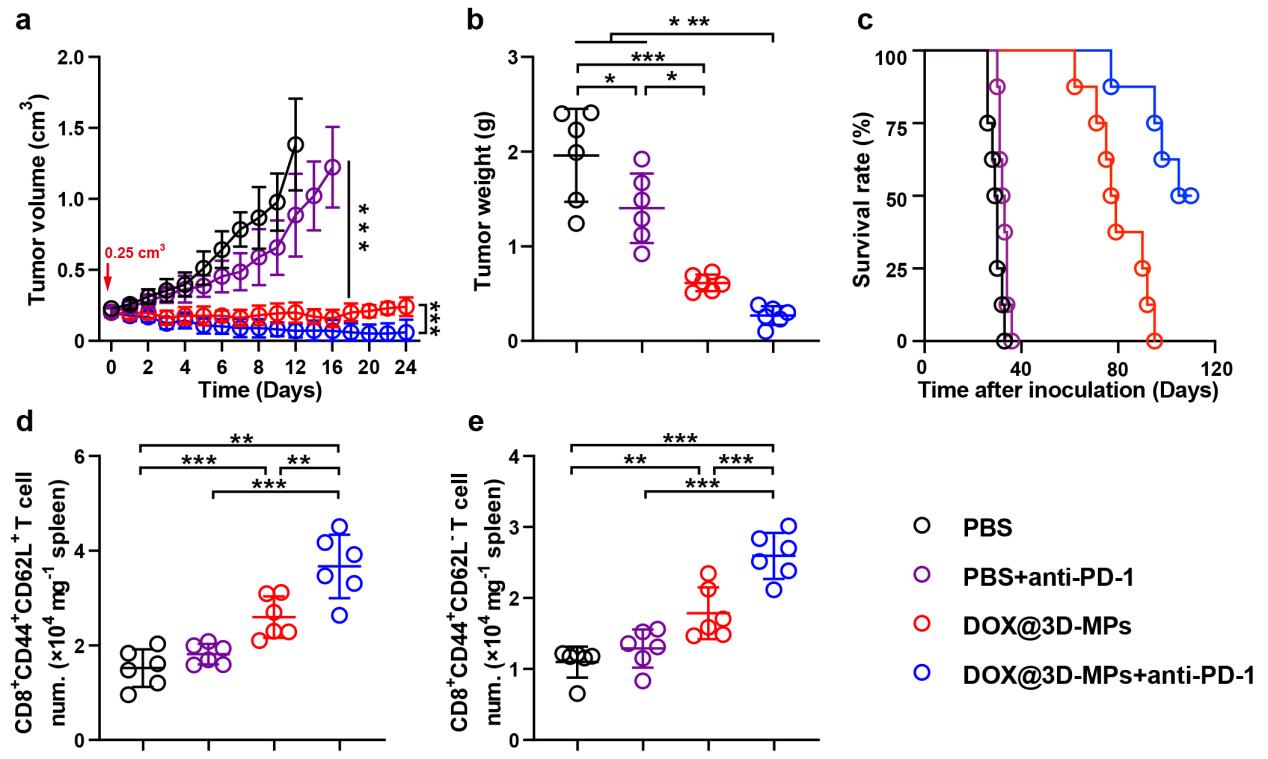
**Supplementary Fig. 27** **Potent antitumor activity and improved immune memory by the combination of DOX@3D-MPs and anti-PD-1 antibody in subcutaneous large H22 tumor-bearing mice.** **a** Tumor growth curves of large H22 tumor-bearing mice after intravenous injection of PBS or DOX@3D-MPs derived from H22 TRCs at DOX dosage of 0.75 mg kg^−1^ once every other day for 6 times in the presence or absence of intraperitoneal injection of anti-PD-1 antibody at dosage of 5 mg kg^−1^ every four days for 3 times. Data are presented as means ± s.d. (n = 8 mice per group). **b** Tumor weights of large H22 tumor-bearing mice at 11 days after treatments indicated in **a**. Data are presented as means ± s.d. (n = 6 mice per group). **c** Kaplan-Meier survival plots of large H22 tumor-bearing mice after treatments indicated in **a** (n = 8 mice per group). **d, e** Numbers of CD8^+^CD44^+^CD62L^+^ T (**d**) and CD8^+^CD44^+^CD62L^-^ T (**e**) cells in the spleens of large H22 tumor-bearing mice at 11 days after treatments indicated in **a** by flow cytometry. Data are presented as means ± s.d. (n = 6 mice per group). **P* < 0.05, ***P* < 0.01, ****P* < 0.001.


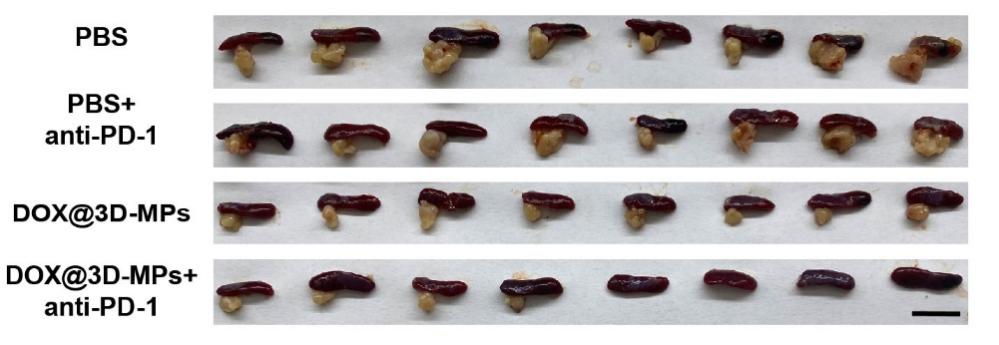


**Supplementary Fig. 28** Images of pancreatic tumors of orthotopic Panc02 tumor-bearing mice at 11 days after intravenous injection of PBS or DOX@3D-MPs derived from Panc02 TRCs at DOX dosage of 0.75 mg kg^−1^ once every other day for 6 times in the presence or absence of intraperitoneal injection of anti-PD-1 antibody at dosage of 5 mg kg^−1^ every four days for 3 times. Scale bar: 10 mm.


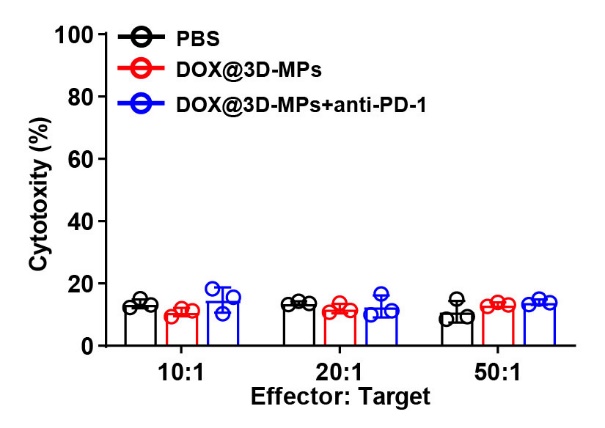


**Supplementary Fig. 29.** Cytotoxicity of T cells (effector cells) against 4T1 cells (target cells) at 24 h after T cells isolated from H22 cell debris-restimulated splenocytes of naive mice, DOX@3D-MPs- or DOX@3D-MPs+anti-PD-1 antibody-cured H22 tumor-bearing mice treated as indicated in Fig. **7a** were incubated with 4T1 cells at different effector/target ratios by LDH assay. Data are presented as means ± s.d. (n = 3 biologically independent samples).


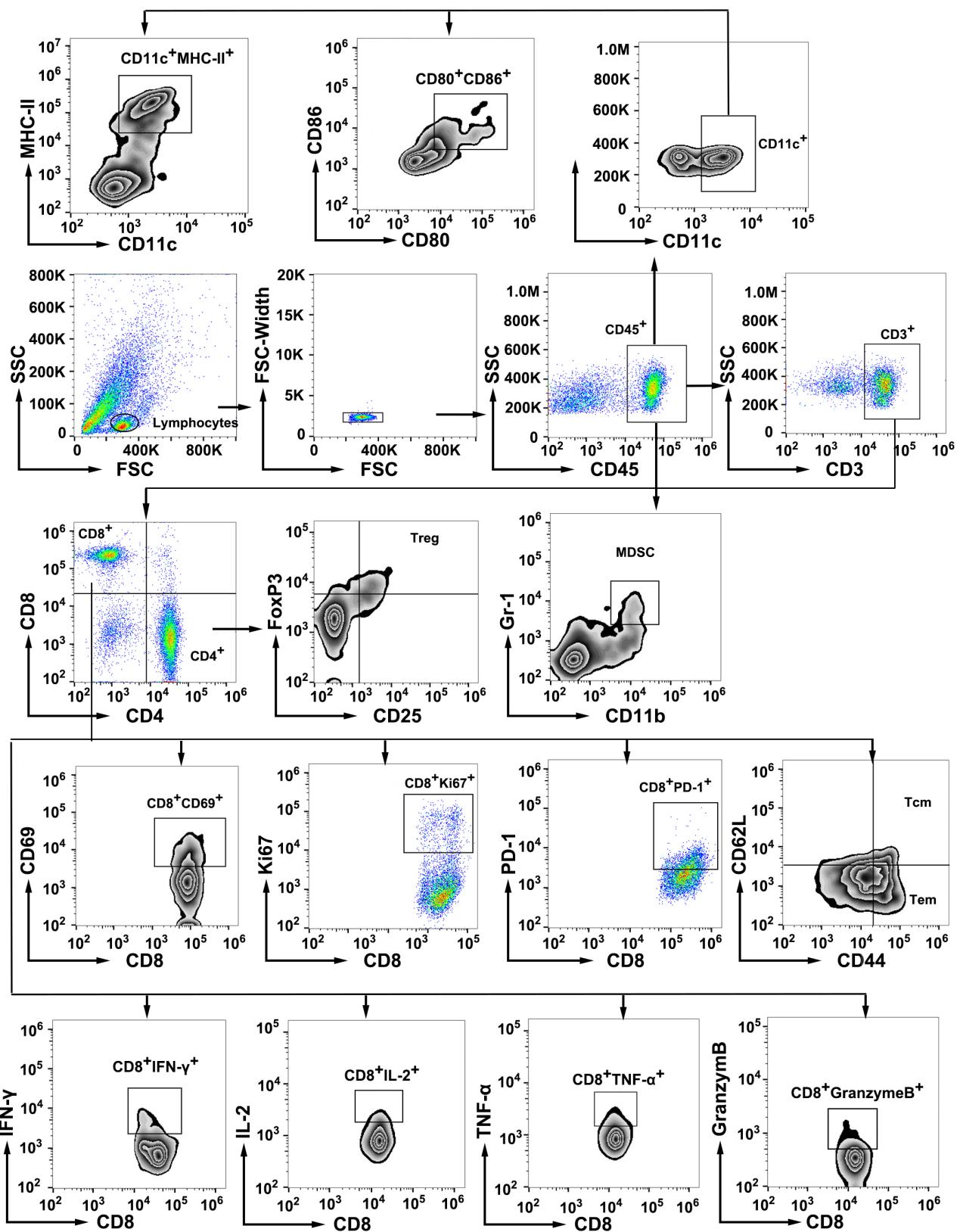


**Supplementary Fig. 30** Gating strategies for identifying matured DCs (CD45^+^CD11c^+^MHC-II^+^ and CD45^+^CD11c^+^CD80^+^CD86^+^ cells), CD4^+^ T cells (CD45^+^CD3^+^CD4^+^ T cells), MDSCs (CD45^+^CD11b^+^Gr1^+^ cells), Tregs (CD45^+^CD3^+^CD4^+^CD25^+^FoxP3^+^ cells), CD8^+^ T cells (CD45^+^CD3^+^CD8^+^ T cells), CD8^+^CD69^+^ T cells (CD45^+^CD3^+^CD8^+^CD69^+^ T cells), CD8^+^Ki67^+^ T cells (CD45^+^CD3^+^CD8^+^Ki67^+^ T cells), CD8^+^PD-1^+^ T cells (CD45^+^CD3^+^CD8^+^PD-1^+^ T cells), CD8^+^IFN-γ^+^ T cells (CD45^+^CD3^+^CD8^+^IFN-γ^+^ T cells), CD8^+^IL-2^+^ T cells (CD45^+^CD3^+^CD8^+^IL-2^+^ T cells), CD8^+^TNF-α^+^ T cells (CD45^+^CD3^+^CD8^+^TNF-α^+^ T cells) CD8^+^GzmB^+^ T cells (CD45^+^CD3^+^CD8^+^GzmB^+^ T cells), Tem cells (CD45^+^CD3^+^CD8^+^CD44^+^CD62L^-^ T cells), Tcm cells (CD45^+^CD3^+^CD8^+^CD44^+^CD62L^+^ T cells) in tumor tissues, lymph nodes or spleens of tumor-bearing mice presented in Fig. **4b-m**, **6e-m**, **7j-n** and Supplementary Fig. **13**-**18**, **23-25**.
